# Understanding ion-transfer reactions in silver electrodissolution and electrodeposition from first-principles calculations and experiments†

**DOI:** 10.1039/d3sc05791g

**Published:** 2024-02-28

**Authors:** Richard Kang, Yang Zhao, Diptarka Hait, Joseph A. Gauthier, Paul A. Kempler, Kira A. Thurman, Shannon W. Boettcher, Martin Head-Gordon

**Affiliations:** a Kenneth S. Pitzer Center for Theoretical Chemistry, Department of Chemistry, University of California Berkeley California 94720 USA mhg@cchem.berkeley.edu richard.kang@berkeley.edu; b Chemical Sciences Division, Lawrence Berkeley National Laboratory Berkeley California 94720 USA; c Department of Chemistry and Biochemistry, the Oregon Center for Electrochemistry, University of Oregon Eugene Oregon 97403 USA; d Department of Chemical Engineering, Texas Tech University Lubbock TX 79409 USA; e Department of Chemical & Biomolecular Engineering, Department of Chemistry, University of California Berkeley California 94720 USA, boettcher@berkeley.edu; f Energy Storage and Distributed Resources Division, Lawrence Berkeley National Laboratory Berkeley California 94720 USA

## Abstract

The electrified aqueous/metal interface is critical in controlling the performance of energy conversion and storage devices, but an atomistic understanding of even basic interfacial electrochemical reactions challenges both experiment and computation. We report a combined simulation and experimental study of (reversible) ion-transfer reactions involved in anodic Ag corrosion/deposition, a model system for interfacial electrochemical processes generating or consuming ions. With the explicit modeling of the electrode potential and a hybrid implicit–explicit solvation model, the density functional theory calculations produce free energy curves predicting thermodynamics, kinetics, partial charge profiles, and reaction trajectories. The calculated (equilibrium) free energy barriers (0.2 eV), and their asymmetries, agree with experimental activation energies (0.4 eV) and transfer coefficients, which were extracted from temperature-dependent voltage-step experiments on Au-supported, Ag-nanocluster substrates. The use of Ag nanoclusters eliminates the convolution of the kinetics of Ag^+^_(aq.)_ generation and transfer with those of nucleation or etch-pit formation. The results indicate that the barrier is controlled by the bias-dependent competition between partial solvation of the incipient ion, metal–metal bonding, and electrostatic stabilization by image charge, with the latter two factors weakened by stronger positive biases. We also report simulations of the bias-dependence of defect generation relevant to nucleating corrosion by removing an atom from a perfect Ag(100) surface, which is predicted to occur *via* a vacancy-adatom intermediate. Together, these experiments and calculations provide the first validated, accurate, molecular model of the central steps that govern the rates of important dissolution/deposition reactions broadly relevant across the energy sciences.

## Abstract 2

Density functional theory simulations and potential-step experiments reveal the atomic charge interactions that govern the ion-transfer kinetics at the electrified solution/Ag interface, providing new insights for energy technology applications.

## Graphical abstract

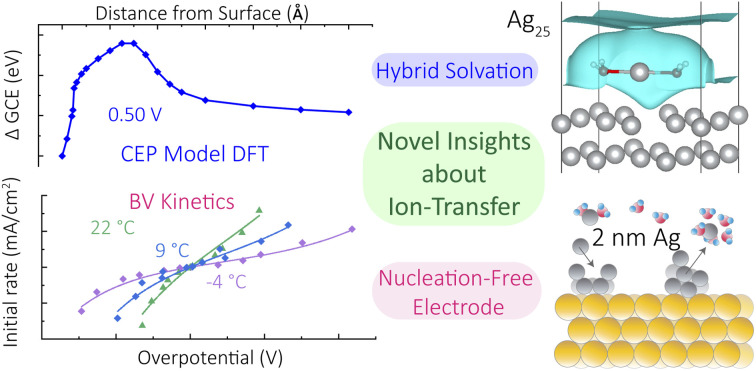

## 1 Introduction

Electrochemical reactions necessarily involve the transfer of both electrons and ions. While there has been tremendous progress developing quantum-mechanical theories of electron transfer,^1,2^ understanding the concomitant ionic reactions is challenged by the molecular complexity of electrified interfaces. A fundamentally important class of reactions are faradaic processes that involve generation or consumption of ions at solid/solution interfaces. The study of such ion-transfer reactions is difficult due to the atomic/molecular complexity of the solid–liquid interface that comprises the electrochemical double layer.^3^ There is no analytical theory that captures the complex behavior at this interface to predict reactivity and rates.^4^ Yet advances in many important applications, such as chemical energy conversion to fuels,^5–9^ durable rechargeable batteries,^10–12^ and corrosion control (estimated to cost 3–4% of global GDP),^13^ could greatly benefit from fundamental understanding of these interfacial reactions and their microscopic mechanisms, which will provide insights on how to control their rates, selectivity, stability, and/or activity.

Fundamentally, even the molecular details of the simplest reactions, *i.e.*, anodic dissolution and deposition involving solvated monovalent cations such as Ag^+^ have been described as an “enigma”.^14^ The factors that control the reaction barriers for the simplest cases, let alone for divalent species like Zn^2+^ and other technologically relevant rare-earth cations,^15^ are surprisingly less studied. To fill this gap, our work here focuses on a model system, the chemically reversible dissolution/deposition of Ag:

1 Ag^+^_(aq.)_ + e^−^_(electrode)_ ↔ Ag_(s)_.

Eqn (1) is thought to be very fast,^16^ although the precise rate constant remains unknown. The mechanism for silver corrosion was proposed by Gerischer in the 1950s, who suggested that the slow initiation step of atom movement to a terrace site is followed by facile dissolution.^17^ The fast rate itself however is not intuitive^14,16^ because the hydration energy of Ag^+^ is ∼5 eV and because water adsorbs weakly to the metal surface, a large free energy barrier might be expected due to desolvation at the interface. Local electric fields (important in catalysis generally^18–20^) under anodic (for dissolution) and cathodic (for deposition) applied potentials are surely critical stabilizing factors, but a molecular picture of the nature of the departing/approaching metal species (*i.e.*, charge and solvation state) remains poorly understood. To address these gaps, our work presents computational results that accurately capture the electronic structure free energy changes as a function of ion-surface separation and applied bias, and a new experimental design that enables isolation of ion-transfer kinetics and associated energy barriers.

Experimentally, the measurement of metal deposition/dissolution kinetics, which involve processes that are competing and multiscale, has long posed a challenge.^21–23^ For metal deposition, the mechanism must involve the transformation of ions in solution into adatoms (or if they carry a partial charge, nominally adions) on a metal surface which undergoes at least partial desolvation. Subsequently, the adatoms (adions) integrate through surface diffusion into low-energy sites, *e.g.* kinks or vacancies, where they incorporate into the lattice.^24^ When these low-energy sites are insufficient to accommodate the flux of incoming ions, new islands form *via* nucleation. Because nucleation, diffusion, and ion-transfer occur simultaneously, the extraction of the various kinetic parameters is not generally possible.^22^ Of course by microscopic reversibility, dissolution contains the same steps as deposition, in reverse. Gerischer *et al.*^17,25^ used chronoamperometry to study the kinetics of Ag/Ag^+^ deposition/stripping. They highlighted challenges in isolating the ion-transfer overpotential (and resulting kinetics) from convoluting mass-transfer/diffusion and ohmic overpotentials, as well as complications from nucleation. Because these measurements were performed on a polycrystalline bulk Ag wire of unknown surface structure and microscopic surface area, which also likely varied with time of deposition/etching, it is not possible to extract the intrinsic kinetic parameters for the Ag^+^ transfer step from these older studies. Later, Mehl and Bockris,^26^ and Despic and Bockris,^21^ argued that the rate-limiting step for Ag electrodeposition was controlled by surface diffusion at low overpotential and ion-transfer at high overpotential by fitting polarization curves to a simple analytical model. Larkin and Hackerman^27^ also reported that the Ag (polycrystalline) — AgNO_3_ adatom/adion surface diffusion determines the rate from near-equilibrium faradaic impedance measurements. STM-based studies of Ag single-crystal facets revealed the dynamics of underpotential deposition (UPD), overpotential deposition (OPD), as well as multilayer growth, which has provided a basis for models of nucleation and growth.^28–31^ Liu *et al.* measured Ag^+^ ion deposition onto Ag-disk electrodes, and found slower kinetics than others found in water, although because of complications associated both with the uncertain microscopic structure of the electrodes studied, and the many underlying assumptions implicit in the analytical models used to analyze the resulting voltammograms,^32^ we are hesitant to directly compare to the kinetic parameters here (ESI, Note 6).† Thus, remarkably, there have been no direct measurements of the kinetic parameters and energy barriers specifically for the ion-transfer step, *i.e.*, associated with solvation changes as the ion crosses the double layer to approach/depart from the surface. This represents a substantial knowledge gap.

The multiscale complexity of the solid–liquid interface in both length and time scales prevents first principles simulations of deposition and dissolution in full dimensionality. One tractable approach is to employ classical molecular dynamics (MD) calculations or Monte Carlo (MC) sampling schemes, informed by quantum chemistry calculations as well as experimental data.^16,33–36^ These studies yielded insights into ion-transfer reactions such as underpotential deposition (UPD) of hydrogen on Pt(111),^33^ concentration and anion co-adsorption effects of Cu UPD on Au(100),^35^ corrosion mechanisms,^16^ and comparison of corrosion inhibitors.^34,36^ Quantum modeling of corrosion *via* density functional theory (DFT) has generated accurate Pourbaix diagrams and calculations of bulk properties.^37–43^ Other quantum-based corrosion modeling focused on solvent and applied electric field effects on potential curves for surface-atom removal, using constrained optimizations.^44,45^ A proposed solution to the enigma of fast Ag^+^ dissolution emerged from DFT calculations that identified a long-range, favorable interaction between the atomic 5s orbital and the metal sp band.^16^ Whilst considerable insight has been gained, there remains a significant gap in that these studies omit the role of electrolyte and applied electrode potential which are both central to electrochemical dissolution and deposition.

In this work, we report a combined computational–experimental study of electrochemical Ag anodic dissolution/deposition using, for the first time in the context of ion-transfer reactions, the constant electrode potential (CEP) model for simulations and transient potential-step measurements. The CEP protocol^46,47^ is a form of grand-canonical DFT (GC-DFT)^48^ that iteratively aligns the Fermi level of the metal with the applied electrode potential to allow the electronic structure of the electrode and interface to respond to applied bias. This model is paired with the linearized Poisson–Boltzmann (LPB) implicit solvation model^49,50^ that provides counterions, in addition to the explicit first-solvent-shell waters to account for molecular desolvation/resolvation. The simulations yield free energy profiles for dissolution/deposition of an Ag^+^ ion, and physical insights into the nature of bias and field-driven effects on the barrier. Information on distance-dependent electron transfer, ion desolvation/resolvation and facet dependence is also obtained. The simulations are directly assessed against new experimental Ag deposition/dissolution data from which activation barriers and transfer coefficients were extracted. For these experiments, a new electrode consisting of Ag-nanoclusters supported on Au with a substantial number of Ag surface-defect sites was designed. This new system eliminates the usually rate-limiting step associated with nucleation. By analyzing the kinetics only from the initial current of the time transients, we largely eliminate the impact of surface diffusion and/or mass transfer on the resulting initial current *vs.* overpotential, which are slower and time-dependent processes. To our knowledge, this is the first report of kinetic parameters that isolate the ion-transfer step through system and experiment design. These new data and insights into the bias-dependent barrier to the ion-transfer reaction in Ag deposition/dissolution helps to build a knowledge base that is relevant to corrosion science, as well as the development of more durable electrocatalysts and light absorbers in solar-fuels systems.

## 2 Results and discussion

While we will use the terms corrosion and electrodissolution interchangeably, we emphasize that our work concerns dissolution of Ag(s) under applied electrochemical bias, rather than its kinetically slower degradation under typical open-circuit conditions.

### 2.1 Bias-dependent simulations of adatom dissolution/deposition

**Fig. 1:**
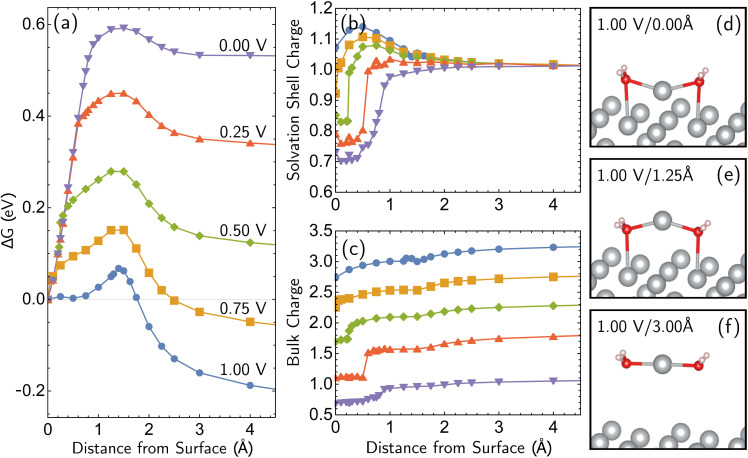
Simulations of adatom (single top-layer Ag atom) corroding from Ag(100) as a function of anode applied bias. (a) Computed change in free energy, Δ*G* with respect to distance from surface of the adatom (all other coordinates optimized for each constrained value of the *z* position of the adatom relative to its equilibrium bound position). Data extends to 6 Å but is not shown. Δ*G* calculated from different references are provided in ESI Note 3B.† (b and c) The partial charge on the adatom and its two first solvent shell water molecules, and the net charge of the remaining surface atoms. The sum of two partial charges is equal to the total charge of the cell for each data point. (d–f) Examples of optimized structures showing the development of the characteristic linear solvent structure associated with the free ion as a function of adatom displacement from the surface.

The most reactive surface atoms are undercoordinated, such as at kinks and steps. A single top-layer atom plays that role in a model closed-packed Ag(100) surface. In brief, the simulation protocol involves constrained geometry optimizations on a periodic unit cell of 25 Ag atoms (24 bulk in 3 layers of 8 atoms, 1 adatom) and two H_2_O molecules, where the *z* coordinate of the adatom (corresponding to the surface normal direction) is fixed. The electrons are self-consistently coupled to a reservoir at the applied potential, leading to loss of electrons at positive bias and partial charges. Although the coordination number of the Ag^+^ ion has been both experimentally^51,52^ and computationally^53,54^ characterized as four, we find that only two explicit H_2_O molecules are necessary to describe Ag^+^ solvation in addition to the linearized Poisson–Boltzmann solvation model, as detailed in ESI Note 2B.† Full details of the periodic DFT approach, the treatment of applied bias, continuum solvent and electrolyte, and the optimizations are given in the Computational details section. The simulations yield bias-dependent free energy profiles for the corrosion/deposition of the constrained adatom, as shown in Fig. 1a. Three illustrations of the resulting optimized structures are shown on the right-hand side (Fig. 1d to f). The top-center panel shows the bias-dependence and distance–dependence of the oxidation state of the departing Ag atom, while the lower-center panel shows how the bulk charge depends on the same variables (Fig. 1b and c).

Focusing on the Δ*G* curve (Fig. 1a), we first note that this simulation yields bias-dependent thermodynamics and kinetics that are qualitatively consistent with experimental information. Regarding thermodynamics, given that the standard reduction potential of Ag is 0.80 V *vs.* SHE,^55^ the favorability of the dissociated Ag^+^ ion described by the free energy curves at positive 1.00 V and 0.75 V compared to the Ag adatom is reasonable. The activation barrier of the corrosion is also qualitatively correct. The activation barrier at 1.00 V *vs.* SHE is 0.068 eV (6.8 kJ mol^−1^), which corresponds to a 298 K turnover frequency (*via* transition state theory) of 4 × 10^11^ s^−1^. This indicates that the reaction is very fast, which is consistent with the experimental observations. The calculated barrier height for 1.00 V *vs.* SHE is also in qualitative agreement with previous Potential of Mean Force (PMF) results of Pinto and co-workers.^16^ We note that the PMF calculation considers the role of electrodes indirectly whereas our CEP calculations consider them explicitly in the free energy. We also note that the turnover frequencies reported may be affected by the entropic contributions from the solvation shell and surface.

Given the unimolecular nature of the reaction, we utilized the Butler–Volmer model^56^ to extract further key characteristics *via*2
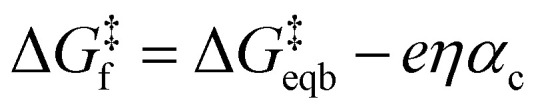
where 
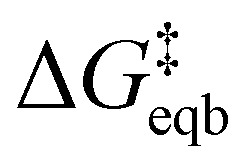
 is the equilibrium activation barrier, *α*_c_ the transfer coefficient, and *η* = *Φ* − *Φ*_eqb_ is the overpotential relative to the equilibrium electrode potential, *Φ*_eqb_. Eqn (2) is, in principle, analogous to the phenomenological Butler–Volmer equation for the exchange current density which will be used later in this work. It is worthwhile to mention the related Brønsted–Evans–Polyani (BEP) model which also posits a linear connection between activation barrier and energy change, and has been extensively used in surface chemistry.^57^ Using a linear fit of the five reaction energies and five activation barriers from each simulated electrode potential, the resulting value of 0.68 V *vs.* SHE for *Φ*_eqb_ was obtained (*R*^2^ = 0.994), which is in reasonable agreement with the experimental value of 0.80 V *vs.* SHE measured for the Ag(100) facet in aqueous solution.^58^ Detailed results and residuals for the linear fits are provided in the ESI.† Indeed a simulated value less than experiment may be sensible given that the simulation is dissolving the last, most ready-to-detach atom on an otherwise perfect surface. The other parameters of eqn (2) that were determined from the fit are 
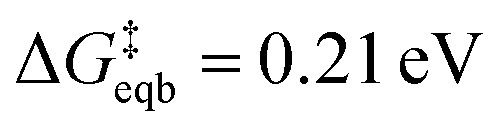
 (*R*^2^ = 0.987) and *α*_c_ = 0.54 (*R*^2^ = 0.995), which will be assessed against experimental data in the following section. In order to provide a reference point for the *Φ*_eqb_ within the computational approach used, we also calculated the bulk reduction potential of Ag(s)/Ag^+^*via* a thermochemical cycle with conventional DFT and obtained 0.82 V *vs.* SHE, in close agreement with the experimental value. (ESI, Note 3A).† The difference of the potentials explored in this work, with respect to this bulk reduction potential, are reported in Table 1.

**Table 1:** Potentials explored in this work, with respect to different reference points^a^

Voltage *vs.* SHE	Voltage *vs. Φ*_bulk,eqb_
1.00 V	0.18 V
0.75 V	−0.07 V
0.50 V	−0.32 V
0.25 V	−0.57 V
0.00 V	−0.82 V

a Calculated bulk Ag redox potential *Φ*_bulk,eqb_: 0.82 *vs.* SHE, theoretical work function of the SHE: −4.43 eV.

What is the molecular origin of the bias-dependent thermodynamics and kinetics discussed above? Within our simulations, the answers for thermodynamics and kinetics are largely the same, because (as seen in Fig. 1a) the change in the forward barrier for anodic dissolution tracks the change in free energy between reactants and products. The strong bias-dependence of the energy change for displacements between 0 and 1.5 Å has several synergistic contributions. Since the 0 → 1.5 Å displacement is breaking the metal–metal bond to the Ag adatom, the first key factor is the weakening of Ag–surface bond by positive applied voltages. The center panels of Fig. 1 (Fig. 1b and c) reveal that the Ag adatom and its two associated H_2_O molecules carry a positive charge at all voltages, but for *z* = 0 that charge is between 0.3*e* and 0.4*e* more positive at 0.75 V and 1.00 V *versus* open circuit. Furthermore, that net charge is slightly larger than +1 at the two most positive voltages. In other words, the adatom complex is pre-oxidized *before* displacement away from the surface at 0.75 V and 1.00 V, partly due to polarization and electron-transfer between Ag(100) and the interfacial waters that solvate the incipient Ag^+^ ion that thus take on H_2_O^*δ*+^ character. By contrast, at the lower voltages, the lower net charge of the Ag adatom is associated with stronger binding to the surface by the Ag adatom. With lower bias, the thermodynamic price of increasing that net charge can no longer be offset by stabilization from partial solvation at the interface.

**Fig. 2:**
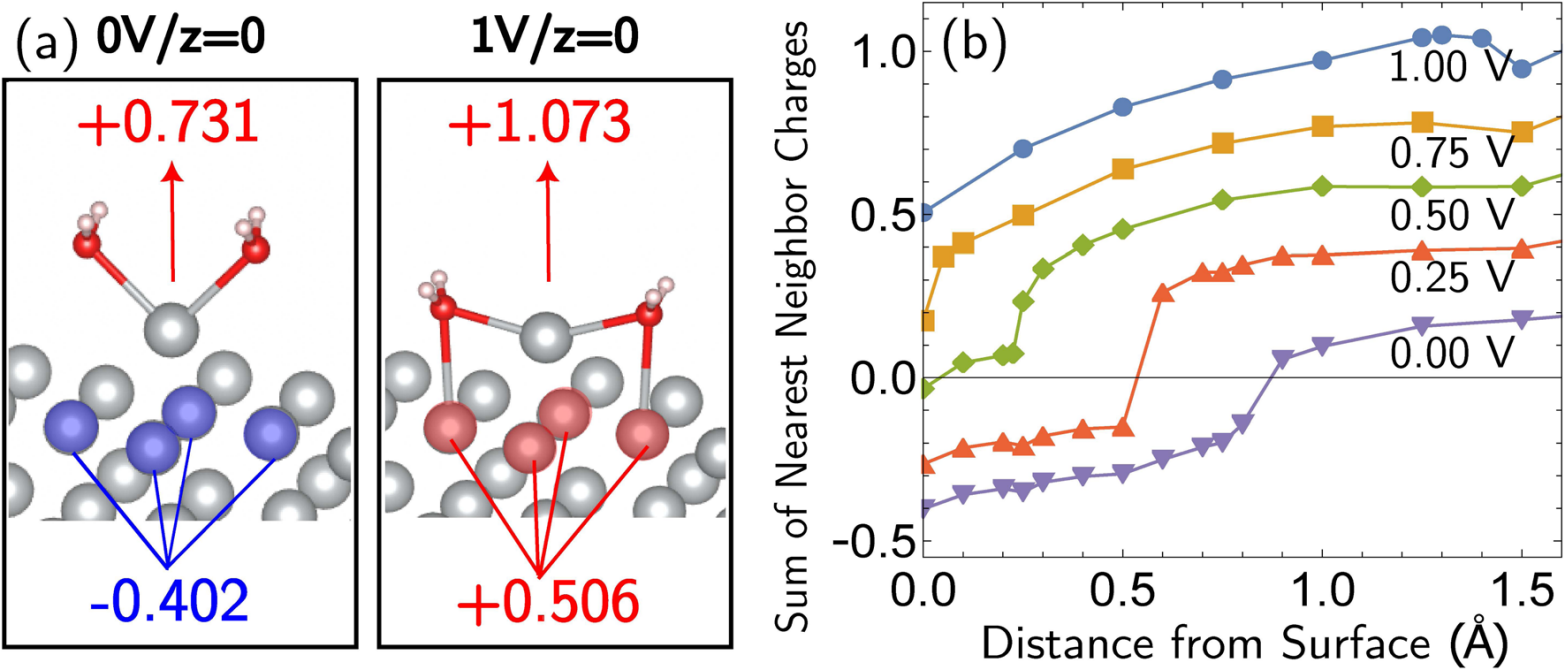
“Image”-type charge interaction in the four surface atoms that are the nearest neighbors of the adatom (same system as Fig. 1). (a) Schematic of the partial charge formation at 0.00 V and 1.00 V optimized (undisplaced) structures of surface plus adatom and two explicit waters, respectively. The top number is the sum of the charges on the adatom and its two waters whereas the bottom number is the sum of the charges on the four nearest neighbor surface Ag atoms. A negative “image”-type charge is formed under the 0.00 V conditions, in contrast to 1.00 V. (b) The dependence of the local surface charge of the four neighbor Ag atoms on the constrained vertical displacement of the adatom, for five different applied biases. These curves show that negative “image”-like character is present at *z* = 0 for 0.00 and 0.25 V. More positive voltages suppress this local negative charge and cause increasingly positive local surface charge.

Beyond loss of binding due to surface charging, there is a second factor strengthening the adatom-bulk interaction for open circuit and low positive biases. As shown in Fig. 2, the simulations predict the formation of a net negative charge on the four surface atoms closest to the Ag adatom for 0.00 and 0.25 V biases, contrary to the clear partial positive charge on the adatom complex even at zero bias, as established on the top central panel of Fig. 1. The opposing signs of the local charges resemble those of the image charges introduced in introductory electrostatics – in order to calculate the field generated by a point charge above an infinite conductor, a point charge with an opposite sign and an equal magnitude is assumed to be induced inside the conductor. The “image”-like charge in our Ag model is similar to this simple example sans the equal magnitude, and this induces an electrostatic attraction between the adatom and the nearest neighbors which must be overcome in order to remove the adatom under zero or low positive bias. The presence of this locally electron-rich Ag atoms is particularly special given that the total charge placed on the cell is positive. This highlights the importance of capturing the quantum mechanical interaction which can influence the microscopic dipole. By contrast, the local surface charge becomes positive at more positive biases, and its electrostatic interaction with the adatom complex becomes repulsive.

The bias-dependence matches our intuition as the more positively charged the surface, the more energetically costly it will be to create a local negative image charge in the solid. Conversely local positive surface charge at corroding potentials is partially stabilized by solvent and electrolyte effects. As also shown in Fig. 2a, molecular water more closely approaches the electrode surface at 1.00 V than 0.00 V, and provides favorable charge–dipole interactions. Similar considerations apply to electrolyte anions (implicitly treated in our simulations).

Additional points can be made on the effects of the molecular solvent along the reaction coordinate for dissolution/deposition. Two solvent molecules plus continuum solvation yield reasonable hydration energies for Ag^+^ (ESI Note 2A, Table S1†), and those molecules coordinate the ion optimally in a linear geometry as shown in the bottom right panel of Fig. 1 (Fig. 1f). By contrast, the surface coordination at *z* = 1.25 Å involves waters closer to the surface than Ag^+^, which then reverses as the adatom binds to the surface to yield a V-shaped coordination geometry. Comparison against a fully implicit model (ESI, Note 2A†) shows that the explicit solvent molecules do not greatly affect the reverse barrier (for deposition), showing that their main role is stabilizing the initial dissociation of the ion. The origin of our calculated corrosion barrier is therefore electronic rearrangements, including the breaking of the adatom–surface bond, and the image–charge interaction at low biases. We note that the free energy change Δ*G* reported in this work is the electrostatic free energy change that includes the contribution from the implicit solvent/electrolyte but not the Gibbs free energy change. In fact, entropic effects associated with the solvent rearrangement (except for the important electrostatic contribution) are not captured in our simulations. It has been previously observed that rigid rotor/harmonic oscillator approximations to add entropic contribution may lead to overestimation of free energy in solvated environments, due to reduced rotational and translational degrees of freedom.^59,60^ In addition, we are mostly interested in the change in the free energy between the initial and final states in this work, which will render the entropic contribution even smaller. Thus we view it as reasonable (as well as necessary for practical reasons) to neglect the entropic contribution for our simulations.

### 2.2 Kinetic measurements of ion transfer in Ag corrosion and deposition

**Fig. 3:**
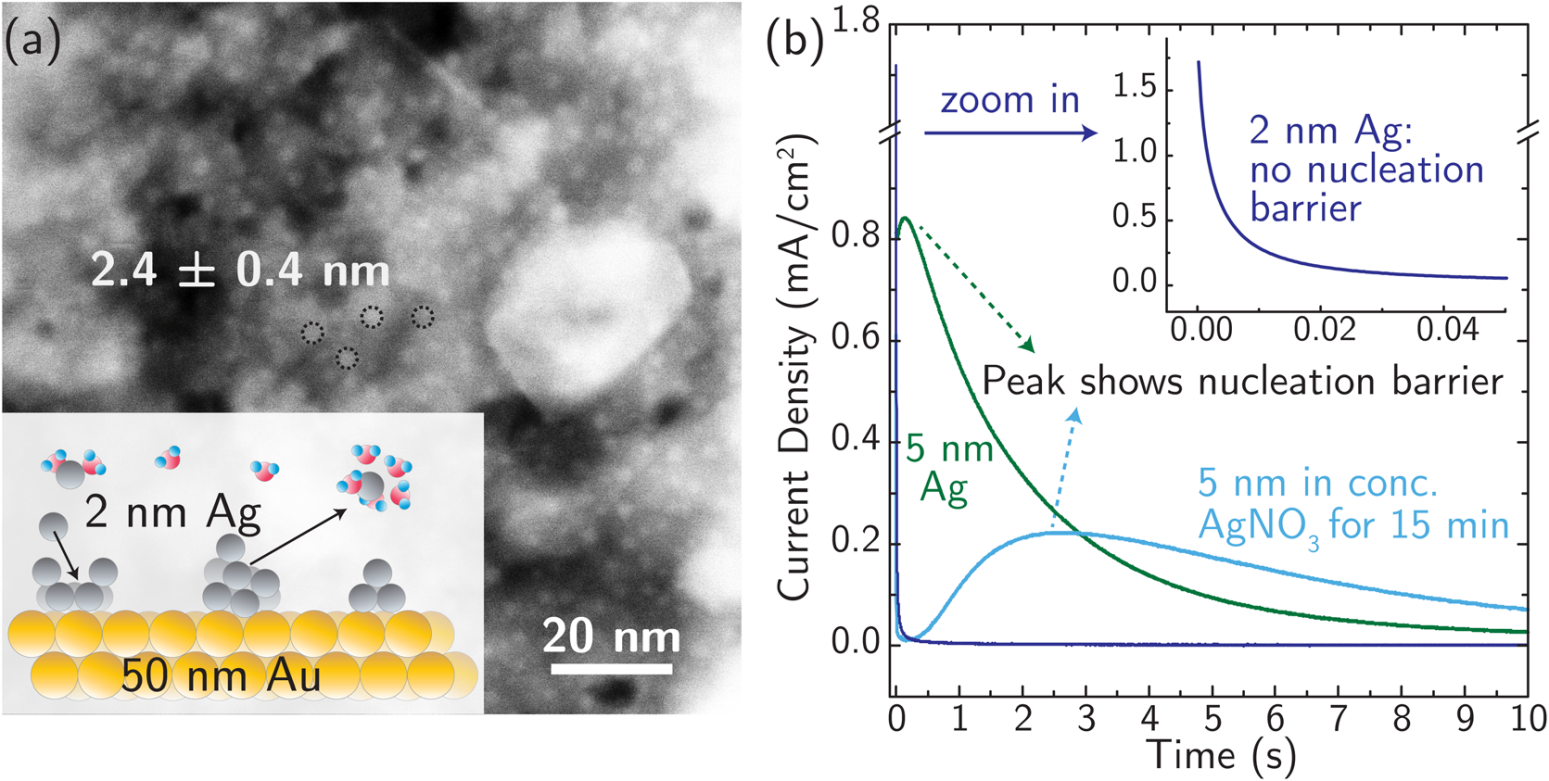
(a) Morphology of the Ag model system with ∼2 nm Ag nanoparticles distributed evenly on a Au substrate (a ∼50 nm thick film), characterized using STEM. The inset is a schematic diagram of the model system. (b) Current transients (*i*–*t* curves) were recorded at 0 °C in 4.7 M AgNO_3_, to avoid concentration overpotentials, by applying constant (over)potential pulses of +50 mV on electrodes with 5 nm Ag and 2 nm Ag nanoparticles, respectively. Note the absence of a peaked current feature associated with nucleation in the 2 nm data.

For simple metals extensively studied experimentally in the past, the electrodeposition rate is typically controlled by the rate of nucleation.^24,61,62^ Similarly, stripping/dissolution is also controlled by the kinetic barrier associated with the formation (*i.e.* nucleation) of etch pits and other high-energy defects. This behavior is captured by the Bewick–Fleishmann–Thirsk (BFT) model,^63^ which explains the peaked current–time profile, like that shown in Fig. 3 for the light blue and green curves, associated with the progressive nucleation of deposition sites after a potential step. In these measurements of metal electrodeposition, ion transfer is fast relative to the rates of nucleation and adatom diffusion such that ion transfer kinetics cannot be resolved.

We designed a new model Ag system to minimize nucleation barriers, and thus enable direct measurement of rate constants associated with ion transfer reactions. Nanosized Ag clusters (nominal film thickness 2–5 nm) on thin, polycrystalline Au films were created using thermal evaporation, as described in the Experimental details section and characterized by high-resolution scanning transmission electron microscopy (HRSTEM) (see Fig. S15†). Fig. 3a shows the schematic design and the morphology of 2.4 ± 0.4 nm Ag nanoparticles well-isolated from each other to provide numerous nucleation active sites for corrosion/deposition processes. The Ag clusters are stabilized by ∼50 nm Au thin film (Fig. S16a†). The presence of the Au support is crucial for stabilizing small, isolated Ag clusters. In the absence of Au, the deposition of 2 nm Ag resulted in the formation of larger aggregates with a size of ∼10 nm (Fig. S16b†).

The presence of small 2 nm Ag clusters is crucial to enable measurements that are not controlled by a nucleation barrier. Fig. 3b shows no nucleation-associated current feature for the 2 nm data and the signature of a nucleation barrier in the initial stage of the transient for the 5 nm Ag data (at ∼0.5 s). After the 5 nm Ag film rests for 15 min in AgNO_3_ electrolyte (light blue curve in Fig. 3b), the peak current occurs at a later time and with a lower peak-current value. This indicates restructuring and aggregation of Ag into larger particles and a reduced number of initial active sites for corrosion. Based on these considerations, 2 nm Ag clusters were used for all subsequent measurements, and all the measurements were taken immediately (see below) after immersing identically and freshly prepared Ag electrodes in the electrolyte (at different temperatures) to avoid reconstruction.

**Fig. 4:**
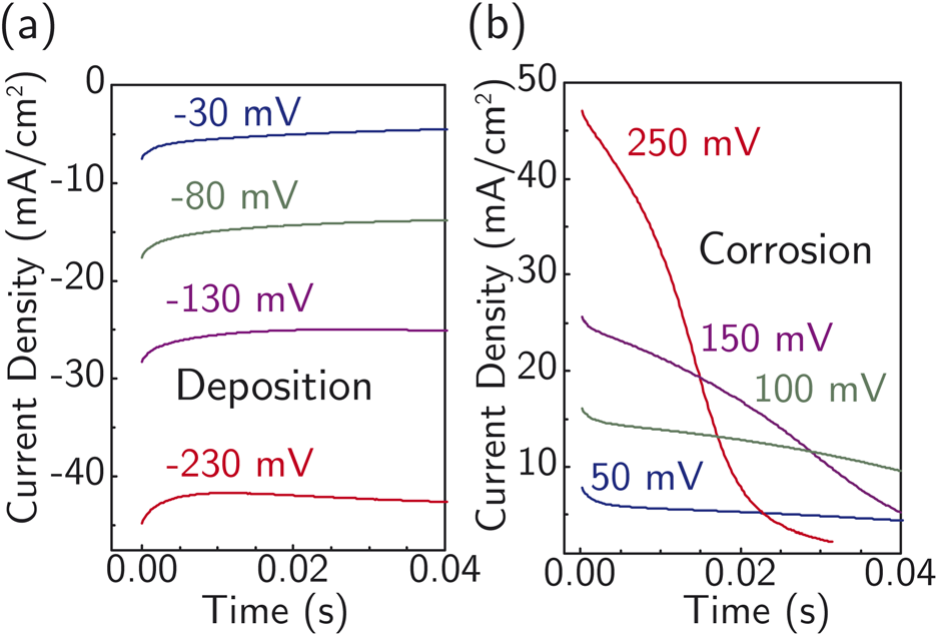
Representative *i*–*t* curves at early times under various constant potential pulses (without *iR* correction from 250 mV to −230 mV) at 22 °C for (a) Ag deposition and (b) corrosion/dissolution without visible nucleation barriers observed. A freshly prepared electrode is used for each measurement.

Fig. 4 displays current transients for the corrosion and deposition of Ag under constant-potential pulses without *iR* correction.^64^ The shape of the transients is largely unaffected by the magnitude of the overpotential, with only the initial current substantially changing. This observation suggests the model design is suitable for studying the ion-transfer kinetics, which we hypothesize controls the initial current magnitude instead of nucleation. The initial current also contains contributions from capacitive charging of the electrical double layer. To approximately correct for capacitive charging, we take the current after waiting *τ* = *RC* where *R* is the cell ohmic resistance and *C* the double-layer capacitance. Impedance measurements (Fig. S17a†) and voltammetry sweeps (Fig. S17b†) in 4.7 M NaNO_3_ were used to estimate *R* and *C* for the system.

On this basis, electrochemical deposition and stripping experiments in a temperature-controlled cell (−4 °C to 33 °C), were carried out to measure ion-transfer rate constants and understand how the apparent activation energy for the Ag^+^ ion transfer during corrosion/deposition depends on the electrochemical driving force. The applied overpotential-pulse values were corrected for uncompensated *iR* to determine the portion of voltage driving ion-transfer kinetics. Measurements were carried out in a random sequence of overpotentials at each temperature. Three replicates were at room temperature were used to establish reproducibility (Fig. S18†).

**Fig. 5:**
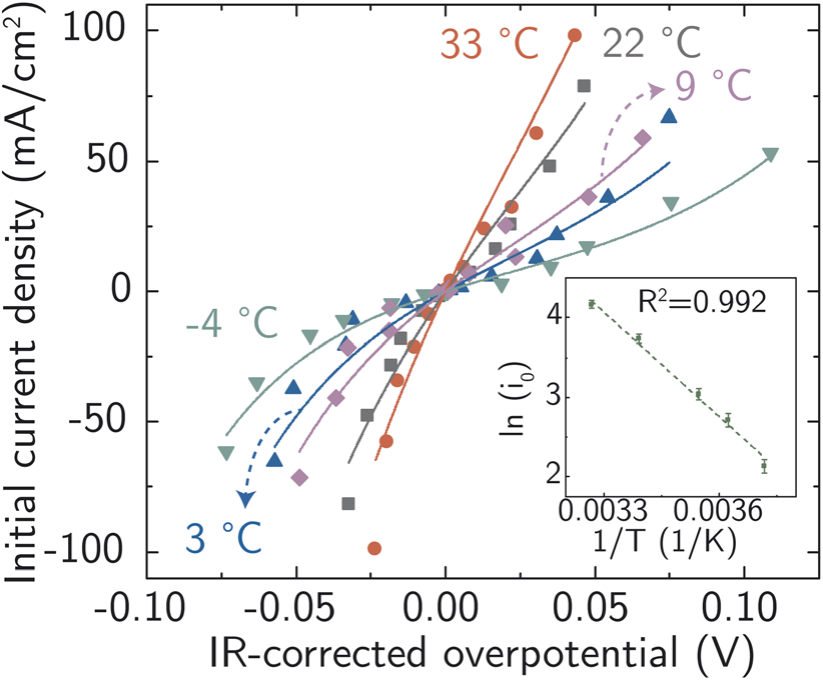
Electrochemical ion-transfer kinetics for Ag-in-water system. Temperature- and driving force (overpotential)- dependent initial rates of Ag corrosion and deposition. The inset shows the resulting Arrhenius analysis used to obtain the activation energy barrier at equilibrium. Each point is a single measurement on a freshly prepared sample.

All the data from Fig. 5 were fit with the phenomenological Butler–Volmer equation to determine the exchange current density *j*_0_:3
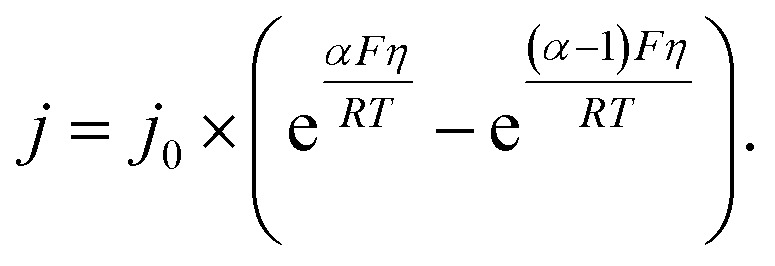


The value of *j*_0_ represents the rate per unit area of the forward and reverse reactions at equilibrium, where we have normalized by the geometric surface area of Ag electrodes. The temperature dependence of *j*_0_ follows the Arrhenius equation 
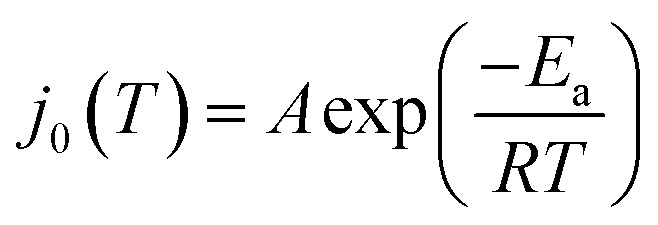
,^24^ where *E*_a_ is the apparent equilibrium activation energy, and *A* is a constant prefactor typically associated with the number of sites, attempt frequency, and entropic factors. Since Δ*G* values of Fig. 1 are electrostatic free energy changes which do not include entropic contributions, *E*_a_ measured by the experiment and 
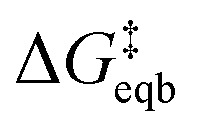
 computed by the simulations can be directly compared. The resulting Ag ion-transfer kinetics exhibits *E*_a_ = 0.38 ± 0.02 eV (36.3 ± 1.9 kJ mol^−1^), along with a transfer coefficient *α* of 0.39 ± 0.02 (Table 2).

**Table 2:** Kinetics parameters extracted from experimental data in Fig. 5

*T* (K)	*i*_0_ (mA cm^−2^)	*R* ^2^ (COD)
269	8.4 ± 0.7	0.98
276	15.1 ± 1.2	0.95
282	20.8 ± 1.5	0.96
295	42.0 ± 2.5	0.97
306	64.2 ± 3.2	0.94
*α*	0.39 ± 0.02	
*E*_a_ (kJ mol^−1^)	36.3 ± 1.9	
*E*_a_ (eV)	**0.38 ± 0.02**	

We also considered the effect of interactions between Ag clusters and the Au substrate that may influence the measured kinetics. First, we note that any cluster-support interaction would be expected to be strongest in the bottom layers of Ag atoms in contact with the Au. Because less than 5% of the Ag atoms in the pristine Ag cluster electrode are dissolved in measuring the initial current for the largest potential step, it is unlikely that Ag atoms in contact with the underlying Au are those dissolving. We also note that the *E*_oc_ of the Au-supported Ag cluster electrode was 8.9 ± 2.2 mV *vs.* an Ag wire, suggesting the free energy of the surface Ag atoms in the cluster is higher than in the polycrystalline Ag. Over time, and with ripening of the Ag nanoparticles, *E*_oc_ returns to approximately 0 mV *vs.* Ag wire. This is consistent with the Ag surface and undercoordinated surface atoms causing the increased energy, not the interaction with the Au substrate. Furthermore, if the kinetics of Ag deposition/stripping were faster on the Au surface than on the Ag cluster, we would not see a largely symmetric current-overpotential response because the open Au area would dominate the current response for negative overpotentials where deposition occurs.

These derived experimental kinetic parameters are reasonably consistent with those from simulation of an adatom on Ag(100) described in the previous section. The DFT-based simulation predicted 
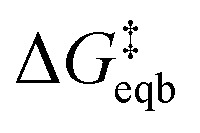
 of 0.21 eV, which is in qualitative agreement with the experimentally measured *E*_a_ value of 0.38 eV, although smaller. Both values are significantly below the 5 eV necessary for monocations to eliminate their hydration shell, showing how cooperative solvation/desolvation, bias-driven local field stabilization, and bond-making/breaking processes control the barriers in corrosion/decomposition at the equilibrium potential. The calculated transfer coefficient of 0.54 also agrees well with the measured value of 0.4, both being close to the typically assumed value of 0.5, and indicating the key role of applied bias in speeding kinetics. Possible factors that could contribute to the underestimation in the DFT-computed values include errors inherent to the exchange–correlation functional and the linearized nature of the solution model, which is partially corrected by the hybrid solvation but may not fully capture the complexity of the double layer. The modeled system is also a simplified chemical surface structure compared to the experimental system where dissolution is surely occurring from a distribution of sites on the surface of the small clusters.

We also experimentally found *E*_a_ at equilibrium of 0.41 ± 0.04 eV in acetonitrile (with 0.1% H_2_O, Fig. S19†) using the same Ag model system. The similarity to the water system is consistent with the mixed explicit water/continuum modeling where much stabilization was due to the explicit water shell. In the experiment, water may still be dominating the direct Ag^+^ solvation even though it is a minority species. It will be interesting to characterize the ion-transfer kinetics in a completely anhydrous MeCN system and other solvent and ligand systems that modulate solvation energy, as well as for different sizes and charges of ions to build a more-comprehensive microscopic picture of the dominate factors controlling these kinetics.

### 2.3 Simulating defect-formation in corrosion

**Fig. 6:**
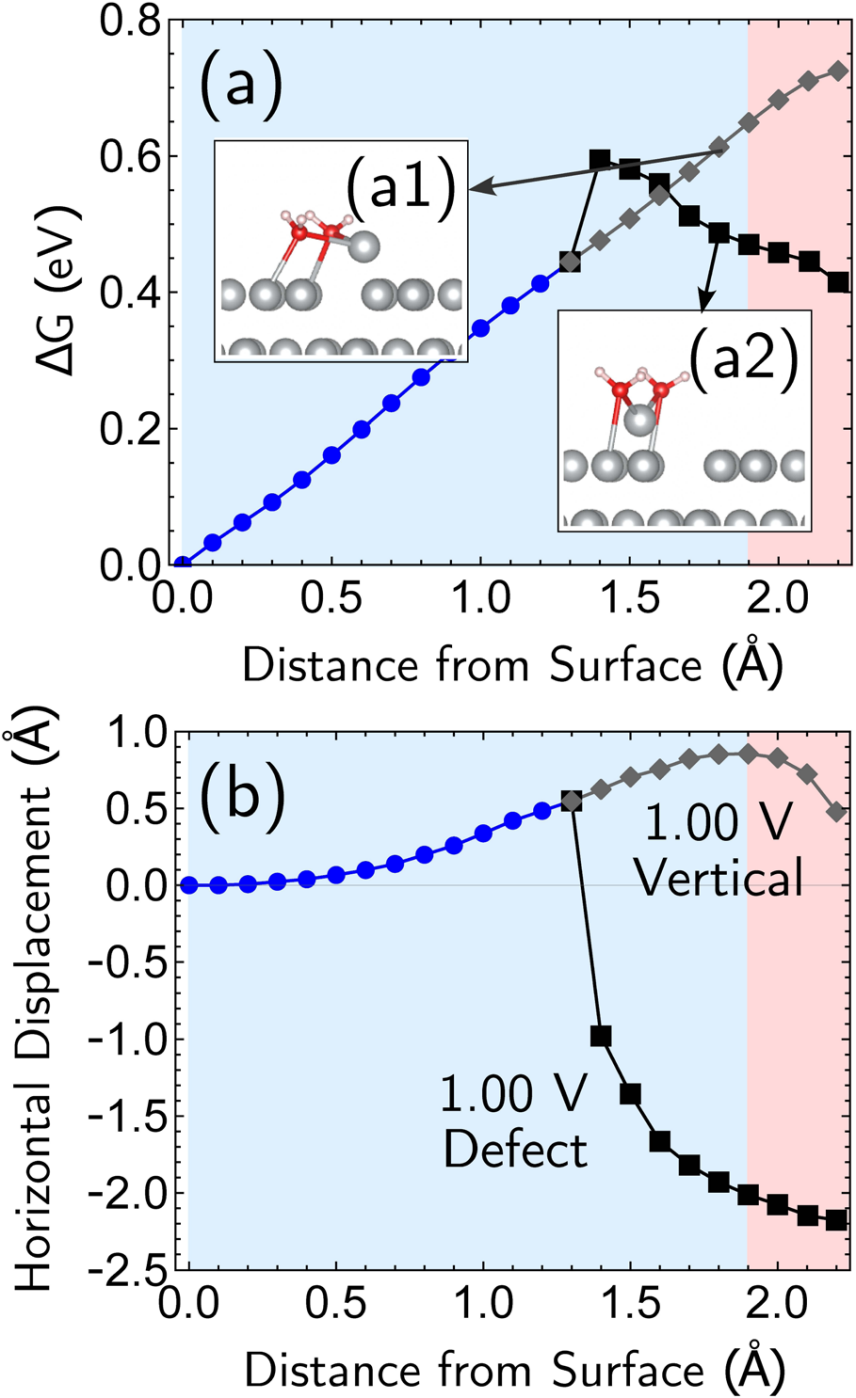
Simulation results for the removal of the first atom from a perfect Ag(100) surface, at 1.00 V *vs.* SHE. The horizontal drift of the departing atom is confined to one dimension, and the drift can be plotted as a signed quantity to indicate the direction. The data reveals that there are two pathways under these conditions, a more favorable pathway that creates a surface defect (adatom) which is subsequently removed, and a less favorable pathway corresponding to direct removal. (a) Two representative structures optimized at 1.7 Å vertical distance from the original position are shown as insets in the top plot of free energy change *versus* displacement of the atom from the surface. Apart from this one constraint, all other geometric parameters were fully optimized. (b) The bottom plot shows the horizontal (in surface plane) displacement for the two paths.

Having established the qualitative accuracy of the simulation protocol against experiment, we next report simulations on different surface morphologies to explore whether there are any interesting changes in the predicted pathways for ion-transfer reactions. To approach the opposite limit of removing/depositing an isolated adatom on the (100) surface, we report the case of a corrosion from a perfect (100) surface. Corrosion from the perfect single-crystal surface should correspond to the most difficult nucleation bottleneck, and we expect far less favorable thermodynamics and kinetics. The results, at 1.00 V *vs.* SHE, in Fig. 6, shows the optimized free energy curve for the single constraint of fixed vertical displacement of one tagged atom, at 1.00 V *vs.* SHE, and the associated optimized value of the displacement in the plane of the surface. Fig. 6 reveals the existence of two possible pathways to create this defect. The first one forms a vacancy–adatom pair structure (*defect-formation pathway*, as illustrated by bottom inset of Fig. 6) from which the adatom subsequently corrodes. In the second pathway, a top layer Ag atom escapes directly into solution (*vertical pathway*) through a bridging structure formed by the waters over the hole. Based on the free energy profile, the *defect-formation pathway* will be less difficult.

**Fig. 7:**
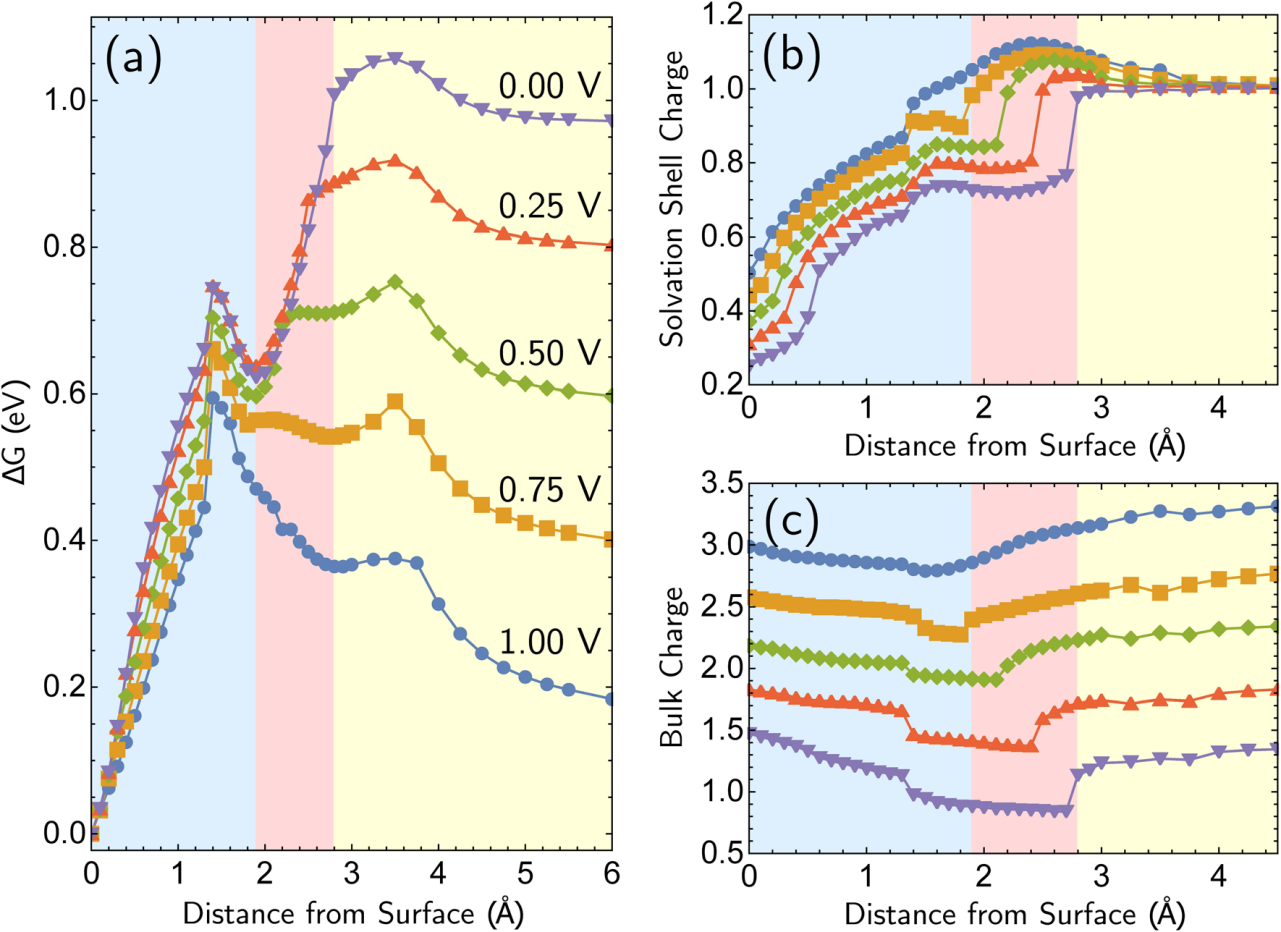
Free energy curves and charge profiles for the *defect-formation* pathway for Ag^+^ formation from perfectly Ag(100). The region approximately between 0 Å to 2 Å denotes the deformation step (blue background), 2 Å to 3 Å the detachment step (pink background), and beyond 3 Å is the adatom corrosion step (yellow background). (a) Free energy curves for the *defect-formation* pathway as a function of applied bias. (b and c) Solvation shell and bulk partial charges as a function of bias for the *defect-formation* pathway. The sum of two partial charges is equal to the total charge of the cell for each data point.

We next consider the bias dependence of the two elementary steps associated with the defect-formation pathway, as shown in Fig. 7. Two activation barriers are obtained. The first one occurs 1.5 Å above the top layer of the surface (blue region, deformation step) where the vacancy–adatom pair is formed (pink region), accessing a local minimum at about 2 Å. This first barrier is only weakly dependent on the applied potential, suggesting that the vacancy–adatom pair formation is governed more by local chemical interaction between metal atoms. This is supported by the fact that the partial charge in the adatom complex is largely insensitive to the applied voltage. The second activation barrier occurs at approximately 3.5 Å above the surface layer (yellow region: adatom detachment regime). This second barrier depends more strongly on applied bias than the first barrier, and exhibits a free energy profile similar to the adatom corrosion case shown previously in Fig. 1. Therefore, we suggest that electrochemically observable corrosion is initiated from already roughened surfaces. This is supported by the fact that the predicted thermodynamics are unfavorable for creation of the adatom defect at 1.00 V in perfect Ag(100) based on the calculations (higher biases change this balance). Partial solvation of the newly created adatom does not fully compensate for its reduced metal–metal bonding strength, even at 1.00 V. Nucleating this defect is a bottleneck, as the chemical process of breaking the bonds of a top layer Ag(100) atom to reform as an adatom are only slightly stabilized by applied bias. Nonetheless, the drop in barrier from 0.75 eV (at 0.00 V) to 0.6 eV (at 1.00 V) makes this nucleation step possible at room temperature. The 298 K, the turnover frequencies associated with these two barriers (calculated through transition state theory) are 1.3 s^−1^ and 4.4 × 10^2^ s^−1^ respectively.

The partial charge analysis shown in Fig. 7 provides interesting data on the role of charge flow in the electrode as a function of Ag atom displacement away from the surface (and as a function of applied bias). The top right plot (Fig. 7b) reveals the generation of the Ag cation as well as some striking effects in the net charge of the remaining surface atoms (“bulk charge”). At low biases, the bulk shows a marked loss of net positive charge at around *z* = 1.4 Å as the barrier to adatom formation is crossed. We associate this with the induction of a negative “image-like” charge in the surface-layer atoms adjoining the partially positively charged adatom (as previously highlighted in Fig. 2) that helps to stabilize the adatom against oxidation. As before, this formation of “image”-like charge is attenuated at higher biases, which synergizes with weakened metal–metal bonding at higher biases to make corrosion more favorable. Notably, the “image”-like interaction is more strongly manifested in the defect-formation pathway compared to the vertical pathway. A comparison of the potential-dependent partial charges for both pathways is shown in ESI, Note 4A.†

We note we have only uncovered a single pathway in the case of a half-etched surface. Interestingly, the qualitative trajectory of the single minimum-energy pathway was dependent on the potential applied (modeled with Ag_28_; ESI, Note 4B).† For a more-positive electrode potential, an Ag atom departing from a step followed an optimal path towards the lower terrace region, whereas at more-negative potentials, the Ag atom drifted towards the higher terrace region to form a geometry similar to a small nanocluster. Subsequently, the final location where the electrochemical dissolution occurred was found to be dependent on the applied potential. This is a consequence of stronger metal–metal interactions for more-negative potentials and stronger metal–water interaction for more-positive potentials. Regardless of the location where the step atom lands during this process, the observation that the atom must first migrate into a terrace site (both in the case of Ag_24_ and Ag_28_) is consistent with the previous observation that diffusion of atoms to terrace sites is an important step to initiate corrosion.^17^

## 3 Conclusions and outlooks

The durability of electrochemical devices presents a challenge that basic science can contribute to by enhancing our knowledge of the elementary steps associated with corrosion and deposition. Despite numerous previous studies, there still exists significant knowledge gaps even for a relatively simple system, such as metallic silver electrodes. In particular, there have been no previous simulations of the bias-dependence of elementary reaction steps such as adatom corrosion/deposition, or the nucleation of a defect site from which subsequent corrosion will be facile. Equally surprising, there are no existing experimental measurements of the ion-transfer kinetics associated with corrosion and deposition that are cleanly separated from other co-existing processes such as nucleation.

We have reported a combined computational and experimental study on silver that fills some of these gaps. On the simulation side, we used density functional theory (DFT) with applied bias, coupled with a mixed explicit/implicit solvent model and continuum electrolyte to explore corrosion/deposition on an Ag(100) surface. This is an advanced model in terms of correctly including the progressive charge depletion associated with the electrode under positive bias, and yielding bias-dependent values for the free energy change for atom corrosion/deposition (eqn (1)), and its associated barrier. The simulation is at the same time highly simplified in terms of accounting only for the minimum explicit solvation needed to account for the hydration energy of Ag^+^, with all remaining solvent and electrolyte effects captured implicitly. Therefore, the simulations omit entropic effects associated with solvent/electrode dynamics. Experimental validation is therefore crucial, and was provided by new measurements that quantify the kinetics of ion-transfer at the electrode interface in the absence of nucleation *via* the use of electrodes comprised of 2 nm Ag clusters deposited on Au supports.

A few of the key results are as follows. For the case of a single adatom on a perfect surface corroding to Ag^+^, the calculated equilibrium potential *vs.* SHE is 0.68 V, with a corresponding kinetic barrier of 0.21 eV, and transfer coefficient of 0.54. Qualitative agreement was observed with our experimental values of 0.38 eV for the kinetic barrier, and a transfer coefficient of 0.4, which serves to broadly validate the simulation approach. Analysis of adatom charge and solvation from the simulations as a function of vertical displacement and applied voltage give insight into the factors promoting corrosion at high positive bias. In brief, these are weakened metal–metal bonding as seen in more positive adatom charge, as well as the inability to develop a stabilizing image charge as occurs at low biases, together with partial solvation of the emerging ion. Simulations were also reported for the case of generating a defect in an otherwise perfect Ag(100) surface that corresponds to a slow nucleation step: the thermodynamics are calculated to be unfavorable even at +1.00 V, and the kinetic barrier is around 0.6 V. The optimal calculated path corresponds to generating a defect–adatom pair, followed by loss of the adatom to solution as an ion. The underlying physical reasons were analyzed. Both the simulation and experimental approach used here can be usefully extended to more heterogeneous and complex processes such as bivalent metal corrosion/deposition, electrolyte and solvent effects, corrosion-related processes at cathodes and more, with substantial opportunity for new insights. In ESI, Note 8†, we discuss how this new strategy developed here to understand elementary ion transfer kinetics might be applied to more reactive metals, like Zn.

## 4 Experimental and computational details

### 4.1 Computational details

#### 4.1.1 Solvation model design

The Q-Chem^65^ package was used to determine the appropriate number of explicit waters. The Polarizable Continuum Model (PCM)^66,67^ (dielectric constant 78.39) was utilized. Starting from the WATER27 (ref. 68 and 69) structures, an Ag cation was coordinated to varying numbers of water molecules. ωB97M-V^70^/def2-SVPD^71,72^ was used for structure optimization, followed by a single-point calculation with def2-TZVPPD^71,72^ for energy calculation. Effective core potentials^73^ were used for Ag. As shown in Table S1 of ESI,† two explicit water molecules is the minimum number needed to adequately reproduce the hydration energy of Ag^+^ in the implicit solvent/electrolyte model. This was then employed for all subsequent corrosion/deposition simulations.

#### 4.1.2 Supercell design

Supercell calculations used Vienna *Ab Initio* Simulation Package (VASP)^74–77^ with the revised Perdew–Burke–Ernzerhof (RPBE) density functional^78^ and the projector augmented wave (PAW) pseudopotentials.^79^ No dispersion corrections were employed, to avoid unphysical overbinding of the corroding atom in implicit solvent/electrolyte (ESI, Note 5).† The lattice constant 4.208 Å for Ag fcc cell was determined through ionic relaxation with variable volume size with a 12 × 12 × 12 Monkhorst–Pack (MP) grid.^80^ The electronic energy and atomic force convergence were 10^−6^ eV and 10^−4^ eV Å^−1^, respectively. A plane-wave cut-off of 600 eV and a Gaussian smearing width of 0.05 eV were used. For the ideal surface (Ag_24_), the Ag(100) supercell contained 3 layers of 8 Ag atoms with a total of 24 atoms, and a continuum solvent/electrolyte of ∼20 Å was placed between two images in the surface normal direction to give a total cell height of 30 Å. This cell size was confirmed through its accurate prediction of the potential of zero charge (PZC). (Experimental PZC of Ag(100): −0.609 V *vs.* SHE,^58^ computed PZC: −0.603 V *vs.* SHE).

The single adatom model (Ag_25_) was constructed starting from this structure. Implicit solvent and electrolyte were modeled *via* VASPsol^49,50,81^ package. Relative permittivity *ε*_b_ = 78.4 and the Debye length *κ*_b_^−1^ = 3.000 Å were used, equivalent to 1.0 M monovalent 1-to-1 electrolyte. The cavitation energy was neglected by setting the surface tension parameter *τ* to 0.000 due to numerical instabilities. The grand canonical free energy obtained included the finite cell-height correction.^82^

#### 4.1.3 CEP calculations

The CEP calculations were carried out with VASP/VASPsol and Atomic Simulation Environment.^83^ The theoretical absolute potential of the SHE *ϕ*_SHE_ was 4.43 eV.^84,85^ The convergence criteria for the electrode potential, electronic energy, and the atomic force was 10^−4^ V (10^−3^ V for challenging cases), 10^−6^ eV, and 0.003 eV Å^−1^ (0.005 eV Å^−1^ for challenging cases), respectively. For the challenging convergence cases, loosened criteria were used and verified against the nearby data points. An MP grid of 6 × 6 × 1 was used. Plane-wave cut-off energy, Gaussian smearing width, and the VASPsol parameters were all identical to that used in the supercell design.

#### 4.1.4 Transition state search

The initial structure was first optimized at *z* = 6 Å from the surface with the optimized two-water solvation shell. Subsequent calculations reduced *z* repeatedly, always starting from the previous converged result, apart from translating Ag(H_2_O)_2_ by the step, *δ*_*z*_ to yield the sequence of constrained optimizations. The procedure was reversed when hysteresis was observed, to get the lower energy structure. Constrained optimizations (selective dynamics in VASP) were utilized — the approaching/departing Ag atom was only allowed to relax in surface parallel direction. All coordinates of the water solvation shell were left free. Due to convergence difficulties, we chose to fix the coordinates of all other bulk Ag atoms at the values from the parent model.

#### 4.1.5 Atomic partial charges

The partial charges were calculated with iterative Hirshfeld (HI) partitioning^86,87^ with the damping function parameter of 1.000. The CEP-optimized structures were used with the final number of electrons.

### 4.2 Experimental details

#### 4.2.1 Chemicals

The electrolytes were prepared from AgNO_3_ (Thermo Scientific, 99.9+%, metals basis), NaNO_3_ (≥99.7%, Honeywell Fluka) and Ultrapure water (Milli-Q gradient, ≥18.2 MΩ cm, TOC < 5 ppb).

#### 4.2.2 Fabrication of Ag electrodes

Glass slides were sonicated for 10 min in acetone (99.8%, Fisher Chemical), iso-propyl alcohol (99.9%, Fisher Chemical), and ultrapure water (18.2 MΩ), followed by O_2_/N_2_ plasma etching. Then, sequentially, 10 nm of Ti at 0.5 Å s^−1^ (electron beam evaporation), 50 nm of Au at 0.5 Å s^−1^ (thermal evaporation), and 2–5 nm of Ag at 0.4 Å s^−1^ (thermal evaporation) were deposited onto the clean substrates at a base pressure below 10^−6^ torr. Following fabrication, the electrodes were stored in a N_2_ and shielded from light until use within 24 h of fabrication.

#### 4.2.3 Electrochemical methods

All electrochemical analyses were carried out in jacketed borosilicate glass cells, equipped with a recirculating chiller (filled with 50/50 volume mixture of propylene glycol and water) to control the cell temperature, with temperature stabilization ensured for a minimum of one hour. The counter and reference electrodes, composed of 99.99% Ag wire, were polished with sandpaper, subsequently submerged in 1 M HNO_3_ (99.99% purity) for 5 min, and then rinsed with ultrapure water prior to use. The glassware was cleaned prior to each experiment by immersion in piranha solution, followed by three sequential rinses with boiling ultrapure water. Immediately prior to each experiment, the electrolytes were purged with N_2_ for ∼20 min. During the measurements, N_2_ was streamed over the cell headspace to prevent air ingress. All electrochemical measurements were carried out using a Biologic (SP-300) potentiostat. Unless stated otherwise, the ohmic drop was corrected for during data analysis. The uncompensated ohmic drop was determined by fitting electrochemical impedance spectroscopy data at open circuit to a Randles equivalent circuit. 4.7 M AgNO_3_ is used as electrolyte throughout the entire work. The open-circuit potential (*E*_oc_) of the Ag cluster working electrode is 8.9 ± 2.2 mV *vs.* Ag wire (reference electrode). For each measurement (with a specific temperature and a specific applied potential), a new Ag cluster electrode is used. To achieve temperature equilibrium, the Ag cluster electrode is placed over the headspace of the jacketed cell for 3 min when the temperature is different from room temperature. Once the new Ag cluster electrode is placed in the electrolyte, the *E*_oc_ and the transient current at applied potential (*η*_a_) are recorded. Subsequently, an impedance measurement was conducted to determine the cell's ohmic resistance *R*, which varied slightly with the placement of the working electrode in the cell and the temperature. The initial current *i*, taken from the *i*(*t*) transient at *t* = *RC* constant was extracted for each potential step experiment, and the corresponding kinetic overpotential (*η*_*k*_) was determined by:*η*_*k*_ = *η*_a_ − *iR* − *E*_oc_

Each data point in Fig. 5 was collected from a single electrode, that is, only one transient was recorded for each pre-prepared model system of Ag nanoclusters on Au, such that each deposition or etching experiment was initiated from an identical initial state with only the driving force overpotential changing between experiments.

## Data availability

The data supporting this article have been uploaded as part of the ESI.† Additional data are available upon request to the authors.

## Author contributions

R. K. and D. H. performed computational work, with assistance and advice from J. A. G. Y. Z. performed experimental work, with assistance and advice from P. A. K. and K. A. T. S. W. B and M. H.-G. supervised the project. R. K. and Y. Z. wrote the manuscript, with inputs from all the authors. All authors reviewed the manuscript.

## Conflicts of interest

The ion solvation calculations were performed in Q-Chem, which is partially owned by M. H.-G. The other authors declare no competing interests.

## Supplementary Material

SC-015-D3SC05791G-s001

SC-015-D3SC05791G-s002

## References

[cit1] Marcus R. A. (1964). Annu. Rev. Phys. Chem..

[cit2] Wörner H. J., Arrell C. A., Banerji N., Cannizzo A., Chergui M., Das A. K., Hamm P., Keller U., Kraus P. M., Liberatore E. (2017). et al.. Struct. Dyn..

[cit3] Helmholtz H. (1853). Ann. Phys..

[cit4] Schmickler W. (2020). J. Solid State Electrochem..

[cit5] Gust D., Moore T. A., Moore A. L. (2009). Acc. Chem. Res..

[cit6] Li J., Wu N. (2015). Catal. Sci. Technol..

[cit7] Montoya J. H., Seitz L. C., Chakthranont P., Vojvodic A., Jaramillo T. F., Nørskov J. K. (2017). Nat. Mater..

[cit8] Zheng T., Jiang K., Wang H. (2018). Adv. Mater..

[cit9] Zhu X., Huang J., Eikerling M. (2021). ACS Catal..

[cit10] Han X., Lu L., Zheng Y., Feng X., Li Z., Li J., Ouyang M. (2019). eTransportation.

[cit11] Cheng X.-B., Zhang R., Zhao C.-Z., Zhang Q. (2017). Chem. Rev..

[cit12] Gao X., Zhou Y.-N., Han D., Zhou J., Zhou D., Tang W., Goodenough J. B. (2020). Joule.

[cit13] Koch G. , Trends in Oil and Gas Corrosion Research and Technologies, Elsevier, 2017, ch. 1, pp. 3–30

[cit14] Gileadi E. (2011). J. Electroanal. Chem..

[cit15] Pinto L. M. C., Quaino P., Santos E., Schmickler W. (2014). ChemPhysChem.

[cit16] Pinto L. M., Spohr E., Quaino P., Santos E., Schmickler W. (2013). Angew. Chem., Int. Ed..

[cit17] Gerischer H., Elektrochem Z. (1958). Ber. Bunsenges. Phys. Chem..

[cit18] Che F., Gray J. T., Ha S., Kruse N., Scott S. L., McEwen J.-S. (2018). ACS Catal..

[cit19] Welborn V. V., Ruiz Pestana L., Head-Gordon T. (2018). Nat. Catal..

[cit20] Dubey K. D., Stuyver T., Shaik S. (2022). J. Phys. Chem. B.

[cit21] Despic A. R., Bockris J. O. (1960). J. Chem. Phys..

[cit22] Sharma S., Zagalskaya A., Weitzner S. E., Eggart L., Cho S., Hsu T., Chen X., Varley J. B., Alexandrov V., Orme C. A., Pham T. A., Wood B. C. (2023). Electrochim. Acta.

[cit23] Boyle D. T., Kong X., Pei A., Rudnicki P. E., Shi F., Huang W., Bao Z., Qin J., Cui Y. (2020). ACS Energy Lett..

[cit24] Fuller T. F. and Harb J. N., Electrochemical Engineering, Wiley, Hoboken, NJ, USA, 1st edn, 2018

[cit25] Gerischer H., Tischer R. P. (1957). Z. Elektrochem..

[cit26] Mehl W., Bockris J. O. (1957). J. Chem. Phys..

[cit27] Larkin D., Hackerman N. (1977). J. Electrochem. Soc..

[cit28] Budevski E., Bostanov V., Staikov G. (1980). Annu. Rev. Mater. Sci..

[cit29] Höpfner M., Obretenov W., Jüttner K., Lorenz W., Staikov G., Bostanov V., Budevski E. (1991). Surf. Sci..

[cit30] Staikov G., Budevski E., Höpfner M., Obretenov W., Jüttner K., Lorenz W. (1991). Surf. Sci..

[cit31] Budevski E., Staikov G., Lorenz W. (2000). Electrochim. Acta.

[cit32] Liu D., Krulic D., Groult H., Fatouros N. (2016). J. Electroanal. Chem..

[cit33] Mateo J. J., Tryk D. A., Cabrera C. R., Ishikawa Y. (2008). Mol. Simul..

[cit34] Guo L., Kaya S., Obot I. B., Zheng X., Qiang Y. (2017). J. Colloid Interface Sci..

[cit35] Weitzner S. E., Dabo I. (2017). npj Comput. Mater..

[cit36] Oukhrib R., Abdellaoui Y., Berisha A., Abou Oualid H., Halili J., Jusufi K., Ait El Had M., Bourzi H., El Issami S., Asmary F. A. (2021). et al.. Sci. Rep..

[cit37] Huang L.-F., Scully J. R., Rondinelli J. M. (2019). Annu. Rev. Mater. Res..

[cit38] Hansen H. A., Rossmeisl J., Nørskov J. K. (2008). Phys. Chem. Chem. Phys..

[cit39] Castelli I. E., Thygesen K. S., Jacobsen K. W. (2014). Top. Catal..

[cit40] Zeng Z., Chan M. K., Zhao Z.-J., Kubal J., Fan D., Greeley J. (2015). J. Phys. Chem. C.

[cit41] Huang L.-F., Rondinelli J. M. (2019). npj Mater. Degrad..

[cit42] Wang Z., Guo X., Montoya J., Nørskov J. K. (2020). npj Comput. Mater..

[cit43] López M., Exner K. S., Viñes F., Illas F. (2022). Adv. Theory Simul..

[cit44] Taylor C. D., Neurock M., Scully J. R. (2008). J. Electrochem. Soc..

[cit45] Taylor C. D. (2009). Chem. Phys. Lett..

[cit46] Goodpaster J. D., Bell A. T., Head-Gordon M. (2016). J. Phys. Chem. Lett..

[cit47] Gauthier J. A., Lin Z., Head-Gordon M., Bell A. T. (2022). ACS Energy Lett..

[cit48] Sundararaman R., Goddard III W. A., Arias T. A. (2017). J. Chem. Phys..

[cit49] Mathew K., Sundararaman R., Letchworth-Weaver K., Arias T. A., Hennig R. G. (2014). J. Chem. Phys..

[cit50] Mathew K., Kolluru V. S. C., Mula S., Steinmann S. N., Hennig R. G. (2019). J. Chem. Phys..

[cit51] Texter J., Hastreiter J. J., Hall J. L. (1983). J. Phys. Chem..

[cit52] Sieradzki K., Dimitrov N., Movrin D., McCall C., Vasiljevic N., Erlebacher J. (2002). J. Electrochem. Soc..

[cit53] Bernasconi L., Blumberger J., Sprik M., Vuilleumier R. (2004). J. Chem. Phys..

[cit54] Weitzner S. E., Pham T. A., Orme C. A., Qiu S. R., Wood B. C. (2021). J. Phys. Chem. Lett..

[cit55] Lide D. R. , CRC handbook of chemistry and physics, CRC Press, 2004, vol. 85

[cit56] Bard A. J. , Faulkner L. R. and White H. S., Electrochemical Methods: Fundamentals and Applications, John Wiley & Sons, 2022

[cit57] Van Santen R. A., Neurock M., Shetty S. G. (2009). Chem. Rev..

[cit58] Trasatti S. and Lust E., Modern Aspects of Electrochemistry, Springer, 1999, ch. 1, pp. 1–215

[cit59] Sumimoto M., Iwane N., Takahama T., Sakaki S. (2004). J. Am. Chem. Soc..

[cit60] Liu C. T., Maxwell C. I., Edwards D. R., Neverov A. A., Mosey N. J., Brown R. S. (2010). J. Am. Chem. Soc..

[cit61] Hölzle M., Zwing V., Kolb D. (1995). Electrochim. Acta.

[cit62] Hölzle M., Retter U., Kolb D. (1994). J. Electroanal. Chem..

[cit63] Bewick A., Fleischmann M., Thirsk H. R. (1962). Trans. Faraday Soc..

[cit64] Zheng W. (2023). ACS Energy Lett..

[cit65] Epifanovsky E. (2021). et al.. J. Chem. Phys..

[cit66] Lange A. W., Herbert J. M. (2010). J. Chem. Phys..

[cit67] Lange A. W., Herbert J. M. (2011). Chem. Phys. Lett..

[cit68] Bryantsev V. S., Diallo M. S., Van Duin A. C., Goddard III W. A. (2009). J. Chem. Theory Comput..

[cit69] Goerigk L., Grimme S. (2011). Phys. Chem. Chem. Phys..

[cit70] Mardirossian N., Head-Gordon M. (2016). J. Chem. Phys..

[cit71] Rappoport D., Furche F. (2010). J. Chem. Phys..

[cit72] Weigend F., Ahlrichs R. (2005). Phys. Chem. Chem. Phys..

[cit73] Andrae D., Haeussermann U., Dolg M., Stoll H., Preuss H. (1990). Theor. Chim. Acta.

[cit74] Kresse G., Hafner J. (1993). Phys. Rev. B: Condens. Matter
Mater. Phys..

[cit75] Kresse G., Hafner J. (1994). Phys. Rev. B: Condens. Matter Mater. Phys..

[cit76] Kresse G., Furthmüller J. (1996). Comput. Mater. Sci..

[cit77] Kresse G., Furthmüller J. (1996). Phys. Rev. B: Condens. Matter Mater. Phys..

[cit78] Hammer B., Hansen L. B., Nørskov J. K. (1999). Phys. Rev. B: Condens. Matter Mater. Phys..

[cit79] Kresse G., Joubert D. (1999). Phys. Rev. B: Condens. Matter Mater. Phys..

[cit80] Monkhorst H. J., Pack J. D. (1976). Phys. Rev. B: Solid State.

[cit81] Mathew K. , Kolluru V. S. C. and Hennig R. G., VASPsol: Implicit solvation and electrolyte model for density-functional theory, 2018, https://github.com/henniggroup/VASPsol

[cit82] Gauthier J. A., Dickens C. F., Ringe S., Chan K. (2019). ChemPhysChem.

[cit83] Larsen A. H., Mortensen J. J., Blomqvist J., Castelli I. E., Christensen R., Dułak M., Friis J., Groves M. N., Hammer B., Hargus C., Hermes E. D., Jennings P. C., Jensen P. B., Kermode J., Kitchin J. R., Kolsbjerg E. L., Kubal J., Kaasbjerg K., Lysgaard S., Maronsson J. B., Maxson T., Olsen T., Pastewka L., Peterson A., Rostgaard C., Schiøtz J., Schütt O., Strange M., Thygesen K. S., Vegge T., Vilhelmsen L., Walter M., Zeng Z., Jacobsen K. W. (2017). J. Phys.: Condens. Matter.

[cit84] Jinnouchi R., Anderson A. B. (2008). J. Phys. Chem. C.

[cit85] Jinnouchi R., Anderson A. B. (2008). Phys. Rev. B: Condens. Matter Mater. Phys..

[cit86] Bultinck P., Van Alsenoy C., Ayers P. W., Carbó-Dorca R. (2007). J. Chem. Phys..

[cit87] Vanpoucke D. E., Bultinck P., Van Driessche I. (2013). J. Comput. Chem..

