# Supplementary material for: Understanding ion-transfer reactions in silver electrodissolution and electrodeposition from first-principles calculations and experiments
Source: Chem Sci. 2024 Feb 28;15(13):4996–5008. doi: 10.1039/d3sc05791g (PMC10966968; doi:10.1039/d3sc05791g)
Supplement: SC-015-D3SC05791G-s002 [file SC-015-D3SC05791G-s002.pdf]

Electronic Supplementary Material (ESI) for Chemical Science.
This journal is © The Royal Society of Chemistry 2024

Electronic Supplementary Information for "Understanding Ion-transfer Reactions in Electrodissolution and Electrodeposition from First-principles Calculations and Experiments"

Richard Kang,[1,2,a)] Yang Zhao,[3] Diptarka Hait,[1,2,b)] Joseph A. Gauthier,[4] Paul A. Kempler,[3] Kira A. Thurman,[3] Shannon W. Boettcher,[3,5,6,c)] and Martin P. Head-Gordon[1,2,d)]

[1)] *Kenneth S. Pitzer Center for Theoretical Chemistry, Department of Chemistry, University of California, Berkeley, California 94720, USA*

[2)] *Chemical Sciences Division, Lawrence Berkeley National Laboratory, Berkeley, California 94720, USA*

[3)] *Department of Chemistry and Biochemistry and the Oregon Center for Electrochemistry, University of Oregon, Eugene, Oregon 97403, USA*

[4)] *Department of Chemical Engineering, Texas Tech University, Lubbock, TX, 79409*

[5)] *Department of Chemical & Biomolecular Engineering and Department of Chemistry, University of California, Berkeley, California 94720, USA*

[6)] *Energy Storage and Distributed Resources Division, Lawrence Berkeley National Laboratory, Berkeley, California 94720, USA*

(Dated: 15 February 2024)

[a)] Electronic mail: richard.kang@berkeley.edu

[b)] Current address: Department of Chemistry and PULSE Institute, Stanford University, Stanford, CA 94305, USA.

[c)] Electronic mail: boettcher@berkeley.edu

[d)] Electronic mail: mhg@cchem.berkeley.edu

CONTENTS

I. Note 1: Theoretical Overview of CEP Model 3
A. Fractional Electrons in KS-DFT 3
B. Constant Electrode Potential (CEP) Model 3

II. Note 2: Design of the Solvation Model 5
A. Fully Implicit Solvation Model 5
B. The Hybrid Solvation Model: Choice of Two Explicit Waters 6

III. Note 3: Supplementary Data for Adatom Anodic Dissolution/Deposition 8
A. Bulk Redox Potential of Ag 8
B. Free Energy Changes plotted against Different References 9
C. Interfacial Capacitance 11
D. Total Charge with respect to the Ion Displacement in Adatom Corrosion 12
E. Turnover Frequency (TOF) 13

IV. Note 4: Supplementary Data for Simulating Defects 14
A. Corrosion from the perfectly-flat surface (Ag_{24}) 14
1. Pathway Dependence of the Partial Charges for Ag_{24} 14
2. Potential Dependence of the Drift in Ag_{24} Corrosion 15
3. Thermodynamics of the Ag_{24} Case 15
B. Corrosion from the half-etched surface (Ag_{28}) 16
1. Free Energy Curves & Partial Charges for Ag_{28} 16
2. Potential Dependence of the Drift in Ag_{28} Dissolution 17
3. Thermodynamics of the Ag_{28} Case 17

V. Note 5: Empirical DFT Dispersion Correction 19

VI. Note 6: Supplementary Note on Comparison with Previous Kinetic Studies 20

VII. Note 7: Supporting Figures for Kinetic Measurement of Ion Transfer in Silver Electrodissolution and Electrodeposition 21

VIII. Note 8: Supplementary Discussion on the Measurement on More Reactive Metals 24

References 25

I. NOTE 1: THEORETICAL OVERVIEW OF CEP MODEL

A. Fractional Electrons in KS-DFT

Many of the quantum chemistry methods consider systems with integer number of electrons, which cannot fully describe inherently grand-canonical, open-systems because fractional electrons represent an averaged behavior. In fact, fractional number formalism and its application in Kohn-Sham Density Functional Theory (KS-DFT) has been actively studied since the birth of its theory.[1,2] To briefly introduce, fractional number can be introduced in occupation number n_i used in electron density

$$\rho(r) = \sum_i n_i |\psi_i(\vec{r})|^2. \tag{1}$$

where $n_i \in [0, 1]$. This allows one to find possible solutions for the corresponding electron density ρ, even for non-integer number values of n_i. Perdew and co-workers showed that the electronic energy of such non-integer number of electrons is exactly determined via a linear interpolation between the nearest two integer electrons energies,

$$E(N - x) = E(N) + x[E(N - 1) - E(N)], \tag{2}$$

where $x \in [0, 1]$ and N is an integer.[2] A conceptually simple understanding of this piecewise-linearity can be obtained from Yang *et al.*[3] In this work, a fractional number electron is explained using a statistical mixture comprised of non-interacting, identical copies. Since the mixing of degenerate, non-interacting do not affect the total energy, the electronic energy of this mixture is thus aligned with the form of Eqn. (2). Study of fractional number problem extends to be very important in the design of approximate KS-DFT,[4] and we recommend interested reader to the appropriate references.

B. Constant Electrode Potential (CEP) Model

Constant Electrode Potential (CEP) model[5,6] makes use of the fractional number formalism described in the previous section. The desired electrode potential with respect to the Standard Hydrogen Electrode (SHE) ϕ_{SHE} can be calculated with the Fermi level ε_F using

$$\Phi = \frac{-\varepsilon_F - \phi_{\text{SHE}}}{e}. \tag{3}$$

In a CEP calculation, the number of electrons is fractionally varied in order to align the Fermi level of the bulk metal with the applied potential bias such that it meets Eqn. (3) to a desired convergence criteria value of $d\Phi$. In practice, this procedure is implemented through adding an extra step of self-consistent loop, on top of the usual electronic minimization of the Kohn-Sham orbitals followed by the structural relaxation of the molecules. Because the CEP model works via varying the number of electrons, the energy of interest of the model is consequently not the electronic energy of the cell on its own, but the free energy (relevant to the grand potential):

$$G(\Phi) = E(\Phi) - q\Phi \tag{4}$$

where E is the electronic energy, q the total charge of the system, and Φ the applied potential calculated via Eqn. (3). Correction to the finite cell height can also be incorporated in the electronic energy as well[7] which has been reported to important for more accurate energetics.[8] In this work, we have taken this additional correction into account. CEP model is therefore a particular type of Grand-Canonical DFT (GC-DFT).[9,10] We note that CEP model is often employed with implicit solvent model that can provide countercharges to ensure a net neutral unit cell, with the most popular choice being the Polarizable Continuum Model/Linearized Poisson-Boltzmann (PCM/LPB) implicit solvation.

II. NOTE 2: DESIGN OF THE SOLVATION MODEL

The schematic of the unit cells used in this study are shown in Figure 1. The size of the unit cell was chosen based on its accurate estimate of the potential of zero charge (PZC) for Ag(100). The experimental PZC of Ag(100) is reported to be −0.609 V vs. Standard Hydrogen Electrode (SHE)[11], and the computed PZC of the model unit cells used was −0.603 V vs. SHE. Three different surface morphologies were built with this base unit cell, and they are shown in Figures 1b through 1d.

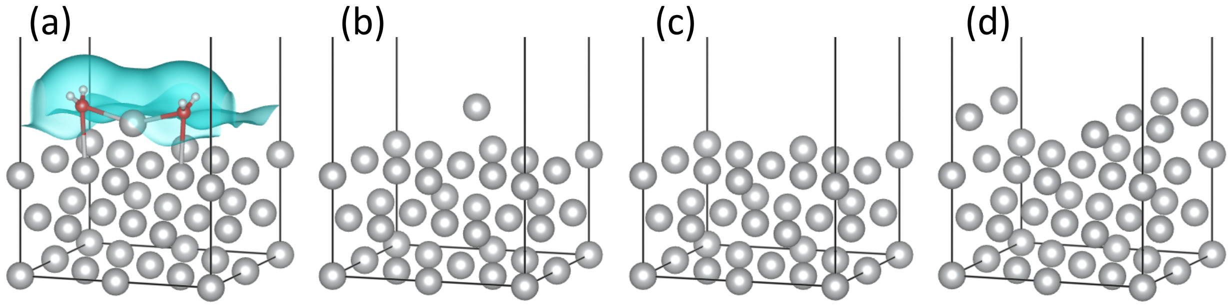

FIG. 1: Schematic of Ag(100) supercells explored in this work. The cell height extends to a total of 30Å for all cases but is not shown. (a) Hybrid implicit and explicit solution representation used in this work. The blue isosurface represents the implicit ionic charge density of -5×10^{-7} eÅ^{-3}. The water molecules around the departing Ag is represented with explicit atoms. (b) Unit cell of the last-atom-bound case (Ag_{25}). (c) Unit cell of perfectly-flat case (Ag_{24}). (d) Unit cell of half-etched case (Ag_{28}). The hybrid solvation representations are used in all of (b-d) but not shown. The graphics are generated with VESTA.[12,13]

A. Fully Implicit Solvation Model

The free energy profile of the fully implicit solvent and electrolyte representation is shown below in Figure 2. The supercell of Ag_{25} was used (Fig. 1b), as the sole adatom bound to the Ag(100) surface would be easiest to corrode out of the three cases. Figure 2 shows that fully implicit description of the electrolyte and solvent successfully captures the generation of Ag^+ but fails to capture the correct thermodynamics of the reaction. On the right panels of Figure 2, the partial charge changes using the iterative Hirshfeld partitioning scheme correctly captures the localized +1 charge in the departing Ag atom for all electrode potentials, while mostly maintaining the bulk charge unchanged. However, corrosion is described thermodynamically unfavorable, even for the highest electrode potential of 1.00 V vs. SHE. Given that the standard reduction potential of Ag is 0.80 V vs. SHE,[14] a potential of +1.00 V is expected to describe the corrosion of an adatom as thermodynamically favored, but the fully implicit description of solution shown by Figure 2 fails to capture this important property. This highlights that the implicit solvation model provides insufficient solvation for ionic species, necessitating further correction to our solvation model.

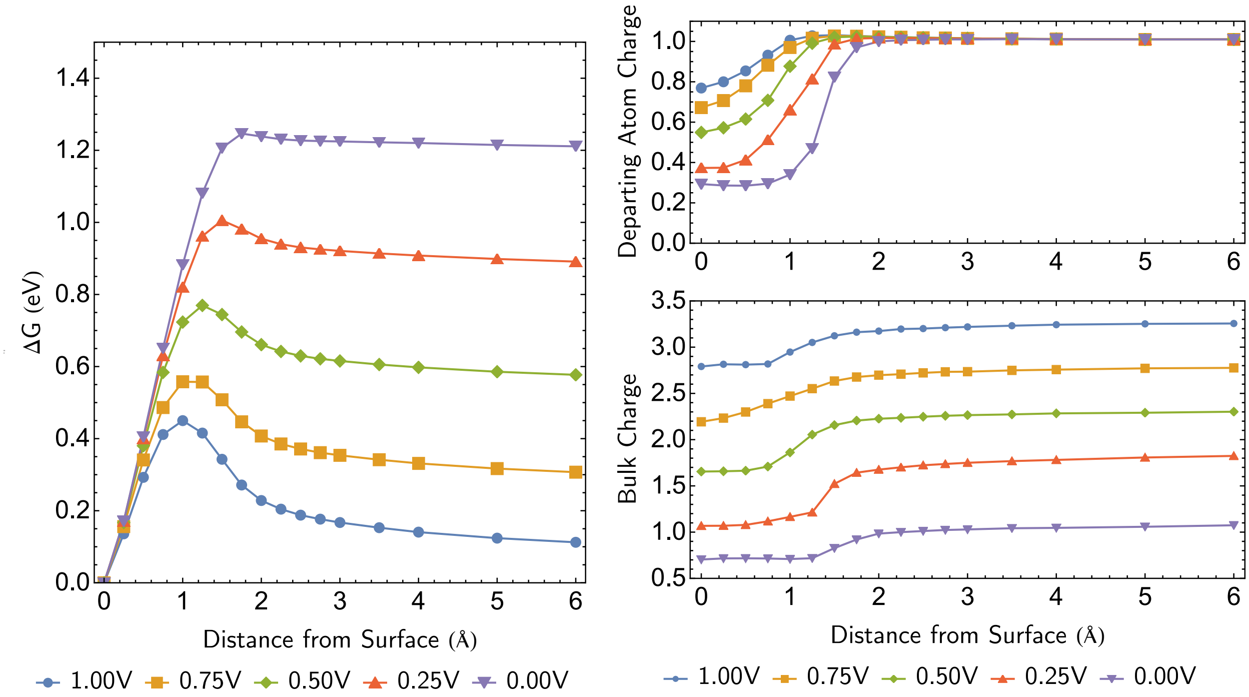

FIG. 2: Last-atom-bound corrosion with fully implicit description of the solvent/electrolyte in Ag_{25}. (*left*) The change in the free energy with respect to the vertical drift of the departing Ag. (*right*) The change of partial charges for the departing Ag and the bulk. The bulk charge is the sum of the partial charges of the 24 Ag atoms in the lattice.

B. The Hybrid Solvation Model: Choice of Two Explicit Waters

In order to obtain a more accurate energy profile, we investigated the most efficient but physical hybrid solvation model required to obtain the correct energetics. We observed that providing sufficient stabilization to the Ag^+ at the dissociated limit would correct the free energies of Figure 2. To this end, we optimized various Ag cation structures with explicit water solvation shell geometries, in the presence of implicit polarizable continuum model (PCM) solvation, to identify any saturation behavior in the hydration energy. Specifically, the formation of Ag^+ solvation shell with n water molecules is described by the reaction

$$[\mathrm{Ag(H_2O)}_n]^+ \rightarrow \mathrm{Ag}^+ + (\mathrm{H_2O})_n, \tag{5}$$

and therefore the energy gain by using n explicit water molecules on top of an implicit solvation is

$$\Delta E^{\mathrm{PCM}}_{\mathrm{hydration}} = \left(E^{\mathrm{PCM}}_{\mathrm{(H_2O)n}} + E^{\mathrm{PCM}}_{\mathrm{Ag^+}} \right) - E^{\mathrm{PCM}}_{\mathrm{[Ag(H_2O)n]^+}}. \tag{6}$$

The results of this calculation are shown in Table I. Noticeably, there is only a minimal variation in the explicit hydration energies after two explicit water molecules, $n = 2$. This means that two explicit waters in the planar geometry ($[\mathrm{Ag(H_2O)_2}]^+$) are sufficient to provide enough stabilization. The total solvation energies, which are calculated by adding the energy difference between Ag^+ in vacuum and Ag^+ in PCM-only (3.481 eV) onto the values of $\Delta E^{\mathrm{PCM}}_{\mathrm{hydration}}$, shows the total solvation energies are qualitatively aligned with the experimentally observed value of 5 eV. We therefore chose to add two explicit water molecules

around the departing Ag^+ cation and used it for CEP calculations. We note that this procedure is can be easily generalized to different solvent systems.

Number of explicit waters (n)	0	1	**2**	3	4	5	6	8
$\Delta E^{\mathrm{PCM}}_{\mathrm{hydration}}$ (eV)		0.583	1.114	1.262	1.172	1.133	1.309	1.066
Total Solvation Energy (eV)	3.481	4.063	4.594	4.743	4.653	4.614	4.789	4.546

TABLE I: Explicit hydration energy $\Delta E^{\mathrm{PCM}}_{\mathrm{hydration}}$ and the total solvation energy for Ag^+ solvation shell with n explicit waters. The total solvation energies for the case of $n \neq 0$ waters are calculated by adding the energy gain using PCM-only (*i.e.*, 3.481 eV) and the corresponding explicit hydration energy $\Delta E^{\mathrm{PCM}}_{\mathrm{hydration}}$.

III. NOTE 3: SUPPLEMENTARY DATA FOR ADATOM ANODIC DISSOLUTION/DEPOSITION

A. Bulk Redox Potential of Ag

The bulk standard reduction potential of Ag was calculated through the following thermochemical cycle:

1. Sublimation Energy: $Ag(s) \longrightarrow Ag(g)$
2. Ionization Energy: $Ag(g) \longrightarrow Ag^{+}(g) + e^{-}$
3. Solvation Energy: $Ag^{+} + 2\,H_2O \longrightarrow [Ag(H_2O)_2]^{+}$ (aq) (+VASPsol)

The sublimation energy of Ag was calculated by constructing a 2x2x2 supercell (5 layers of 8 atoms) of Ag(100) (with the same dimension of the box used in the CEP calculations of this work), and consequently calculating the energy change associated with removing the Ag atom at the center of the third layer and placing it between the two periodic images. The ionization energy of Ag was calculated on a 20 Å × 20 Å × 20 Å box with Ag and Ag^{+} placed on the center. The calculation of Ag^{+} incorporated dipole correction on all directions. For the solvation energy, the optimized $2\,H_2O$ structure, determined in the previous section (while designing the solvation model), was placed in the same 20 Å × 20 Å × 20 Å cubic box. This energy was summed with the energy of the Ag cation and compared against the energy of $[Ag(H_2O)_2]^{+}$ embedded in VASPsol. The calculated energy for each component is tabulated in Table II. All of the calculations employed VASP for consistency with the RPBE functional. Ionization energy and sublimation energy was calculated with Gamma-point only Monkhorst-Pack grid, while the sublimation energy was calculated with $6 \times 6 \times 1$ Monkhorst-Pack grid. Summing each of the components and subtracting by 4.43 eV (the theoretical work function of SHE) results in 0.82 V vs. SHE, which is in very good agreement with the experimentally reported Ag bulk redox potential.

Sublimation Energy			
	2x2x2 supercell	2x2x2 supercell with center Ag displaced	ΔE
Energy (eV)	-82.610643 eV	-79.704438 eV	2.91 eV
**Ionization Energy**			
	$Ag(g)$	$Ag^{+}(g)$	ΔE
Energy (eV)	-0.020988 eV	7.630450 eV	7.65 eV
**Solvation Energy**			
	$Ag^{+} + 2\,H_2O$	$[Ag(H_2O)_2]^{+}$ (aq) (+VASPsol)	ΔE
Energy (eV)	-20.785855 eV	-26.095840 eV	-5.31 eV

TABLE II: Components used in the calculation of bulk Ag standard reduction potential.

B. Free Energy Changes plotted against Different References

Figures 3, 4, and 5 show the calculated free energy changes, calculated with respect to different reference points. The computed energies are given by the following equations:

$$\Delta G_{\text{dissolution}}(V) = G(z,V) - G(z = 0\text{Å}, V) \tag{7}$$

$$\Delta G_{\text{deposition}}(V) = G(z,V) - G(z = 6\text{Å}, V) \tag{8}$$

$$\Delta G[\text{Ag}^+_{(aq)}](V) = G[\text{Ag(s)} + \text{Ag}^+_{(aq)}](z = 6\text{Å}, V) - G[\text{Ag(s)}](V) \tag{9}$$

where the quantity described by Eq. (7) is precisely the one reported in Fig 1 of main text.

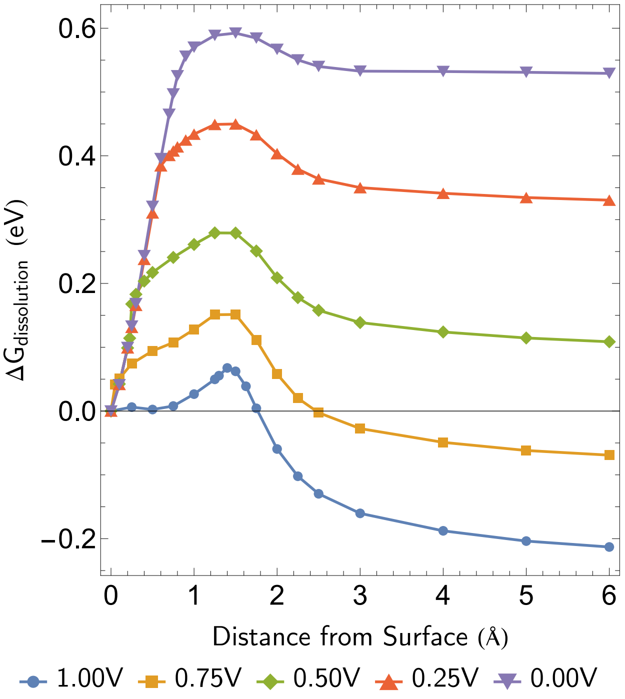

FIG. 3: Potential-dependent $\Delta G_{\text{dissolution}}$, computed with respect to the 0Å structure. Identical to Fig. 1a in main text, but now showing all data points collected.

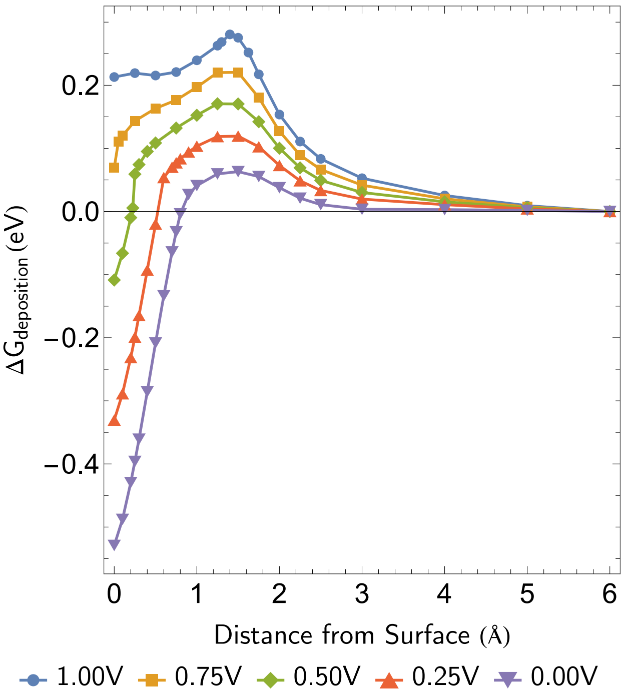

FIG. 4: Potential-dependent $\Delta G_{\text{deposition}}$, computed with respect to the 6Å structure.

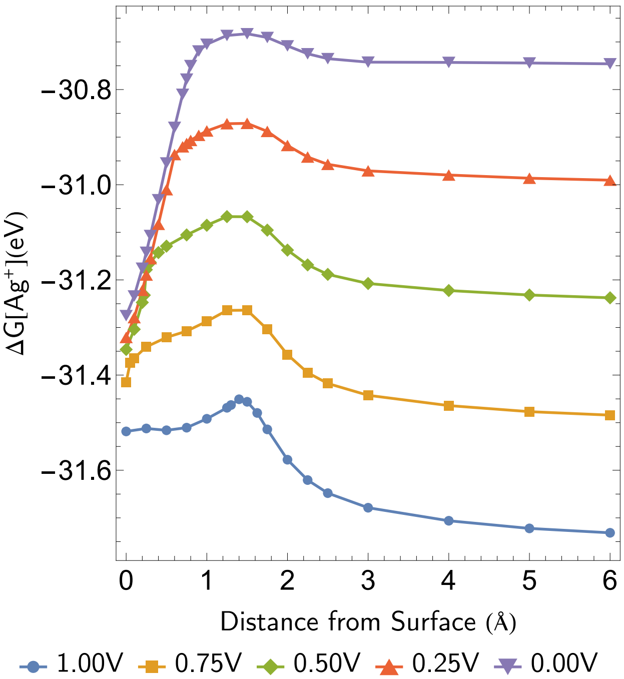

FIG. 5: Potential-dependent $\Delta G[\mathrm{Ag}^{+}_{(aq)}]$. Ag(s) is modeled with 3 layers of 8 Ag atoms, and the free energy is computed with CEP protocol.

C. Interfacial Capacitance

To estimate the effect of two explicit waters on the interfacial capacitance, we used the 1.00 V optimized structure of Ag_{25} at 0.00 Å displacement above surface normal (including the two explicit waters) and varied the charge in the cell to observe the linear response of the voltage and subsequently calculate capacitance via $C = dq/dV$. Although this is a rough approximation, this provides a good ballpark approximation to gauge the effect of two explicit waters. The calculated values as well as the linear fitted slope are tabulated in Table III. The resulting value of the slope is estimated to be 2.033×10^{-19} C/V, and divided by the area of xy-plane of our cell (70.8079 Å^2, both sides of the slab gets charged) results in interfacial capacitance of 0.1436 F/m^2, which is lower than the value of 0.4 F/m^2 experimentally observed,[15] but qualitatively correct by being in the right order of magnitude.

$N_{\text{electrons}}$ (eV)	290.5	290	289.5	289	291.5	292	292.5	293
Charge on cell (e_0)	+0.5	+1.0	+1.5	+2.0	-0.5	-1.0	-1.5	-2.0
Voltage (vs. SHE)	-0.808	-0.457	-0.178	0.107	-1.648	-2.070	-2.560	-2.964
Slope: 2.033×10^{-19} C/V								
Fit Results: $R^2 = 0.9939$								

TABLE III: Calculation of interfacial capacitance.

D. Total Charge with respect to the Ion Displacement in Adatom Corrosion

Figure 6 shows the calculated total charge in the cell for each applied voltage, plotted with respect to the displacement of the ion solvation shell.

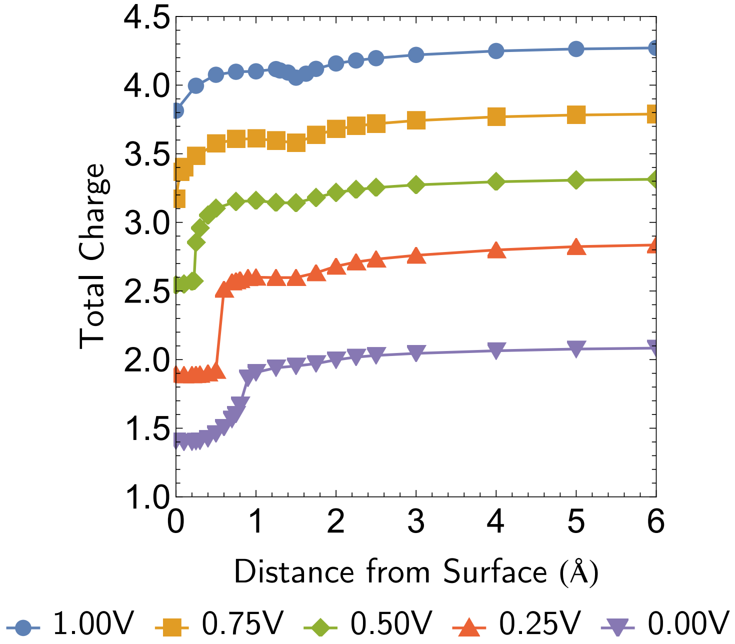

FIG. 6: Calculated total charge of the cell with respect to the ion-displacement in Ag_{25}

E. Turnover Frequency (TOF)

Figures 7 and 8 show the turnover frequency (TOF) of the adatom corrosion (Ag_{25}), plotted against the activation barrier (Fig. 7) and the applied voltage (Fig. 8), respectively.

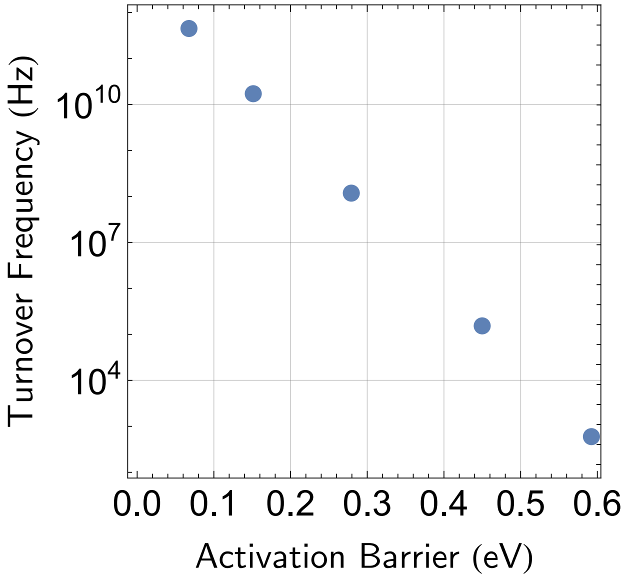

FIG. 7: TOF of the adatom corrosion (Ag_{25}) with respect to the activation barrier.

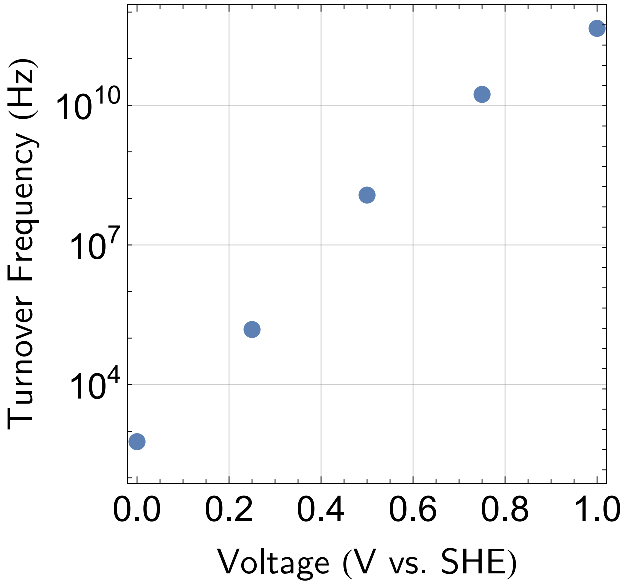

FIG. 8: TOF of the adatom corrosion (Ag_{25}) with respect to the applied voltage.

IV. NOTE 4: SUPPLEMENTARY DATA FOR SIMULATING DEFECTS

This section lists supplemental data relevant to simulating defects in corrosion/deposition which are the perfectly-flat (Ag_{24}) case and the half-etched (Ag_{28}) from the main text.

A. Corrosion from the perfectly-flat surface (Ag_{24})

1. *Pathway Dependence of the Partial Charges for Ag_{24}*

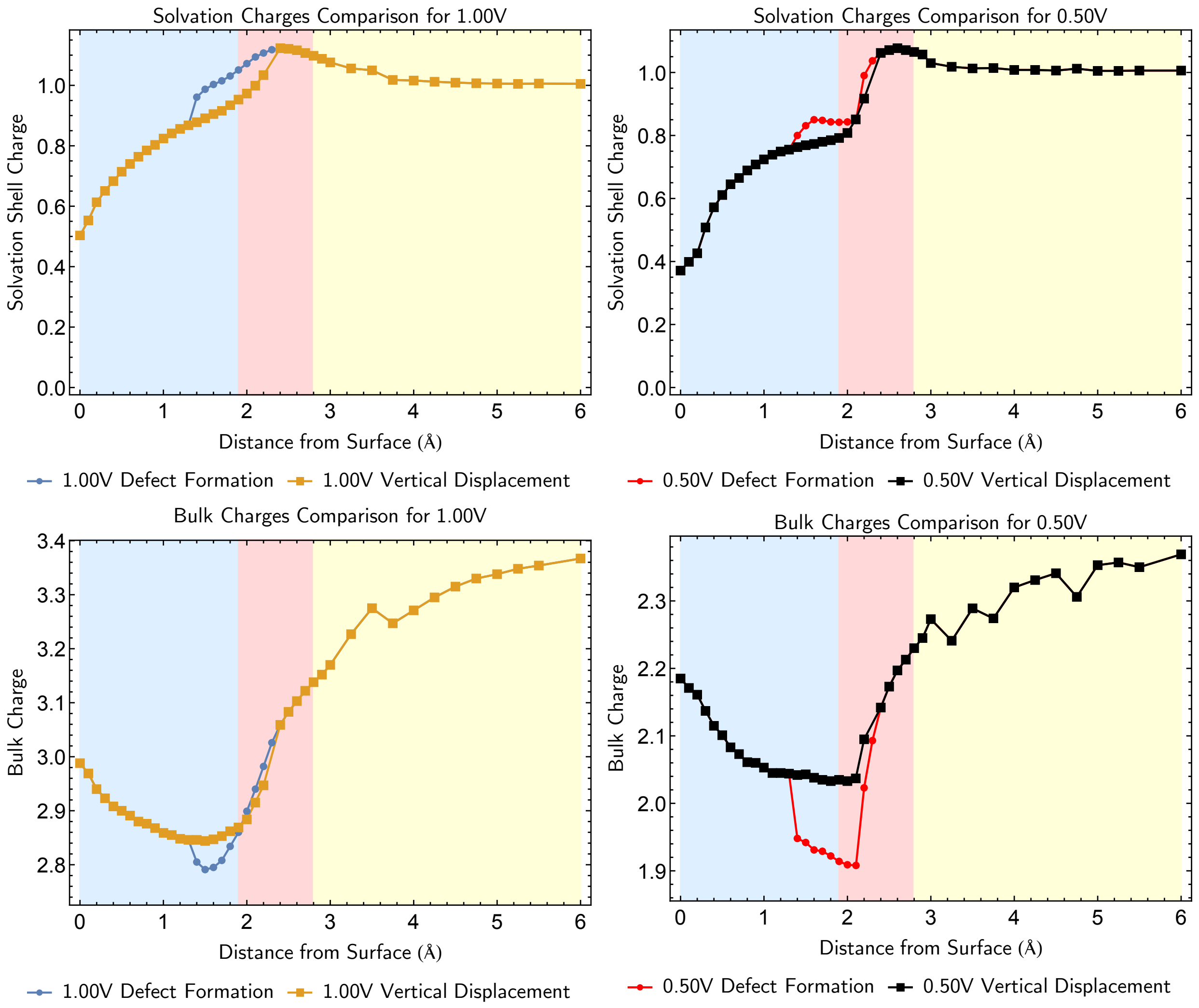

FIG. 9: Potential Dependence of the horizontal drift in defect-formation pathway.

Figure 9 shows that the defect-formation pathway induces more changes in the partial charges in the detachment step (pink region) in the course of corrosion/deposition, both in the solvation shell and in the bulk. This is explained by the horizontal drift associated with the defect-formation pathway, which causes the departing atom to be be placed closer to the Ag(100) surface than the vertical pathway. This induces stronger "image"-type charge interactions between the solvation shell and the bulk.

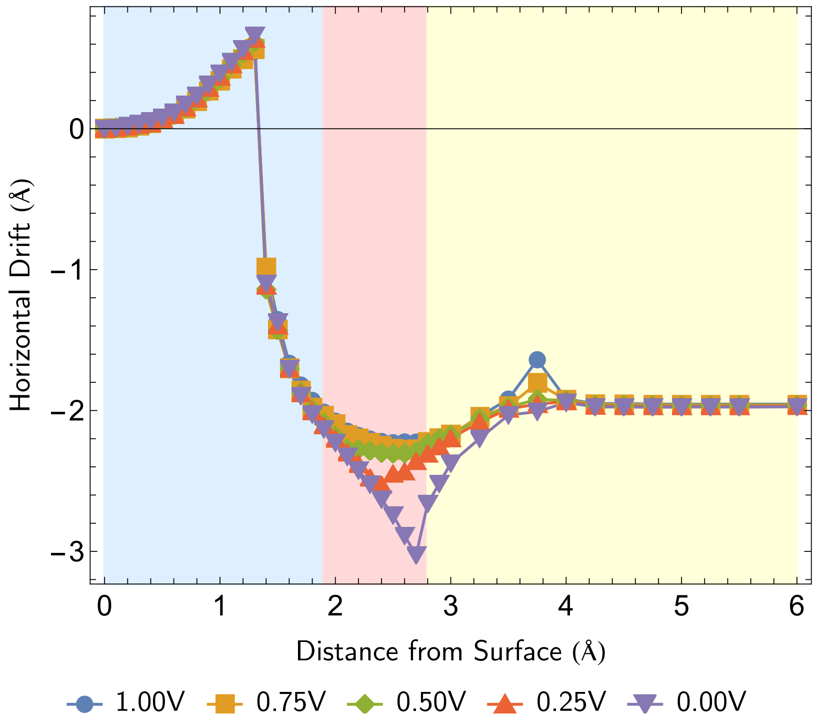

FIG. 10: Potential dependence of the departing solvation shell's horizontal for Ag_{24} defect-formation pathway. Since the horizontal drift is confined to one-dimension, the value can be plotted as a signed to quantity to indicate the direction of the drift. In this plot, a 0 Å horizontal drift represents a vertical displacement above the vacancy position.

2. Potential Dependence of the Drift in Ag_{24} Corrosion

Figure 10 shows that lower potentials result in more horizontal drift fluctuations in the defect-formation pathway of Ag_{24}. This is most evident in the detachment step (pink region) of the Ag24. This can be explained by the stronger metal-metal interaction under the presence of lower potential condition, when compared stronger anodic conditions.

3. Thermodynamics of the Ag_{24} Case

In Table IV, the absolute free energies for uncorroded/corroded structures under strong potentials of 1.25 V and 1.50 V are provided for Ag_{24}. The results show that application of very strong potentials eventually leads a favorable corrosion, assuring the qualitative correctness of the CEP calculations on this model.

Potential (V)	G of Ag_{24} 0 Å structure (eV)	G of Ag_{24} 10 Å structure (eV)
1.25 V	**-80.3541**	-79.4658
1.50 V	-81.3837	**-81.6745**

TABLE IV: **Absolute** free energies (G) of perfectly-flat case Ag_{24} for potentials higher than 1.00 V. Energetically lower points are boldfaced.

B. Corrosion from the half-etched surface (Ag_{28})

1. *Free Energy Curves & Partial Charges for Ag_{28}*

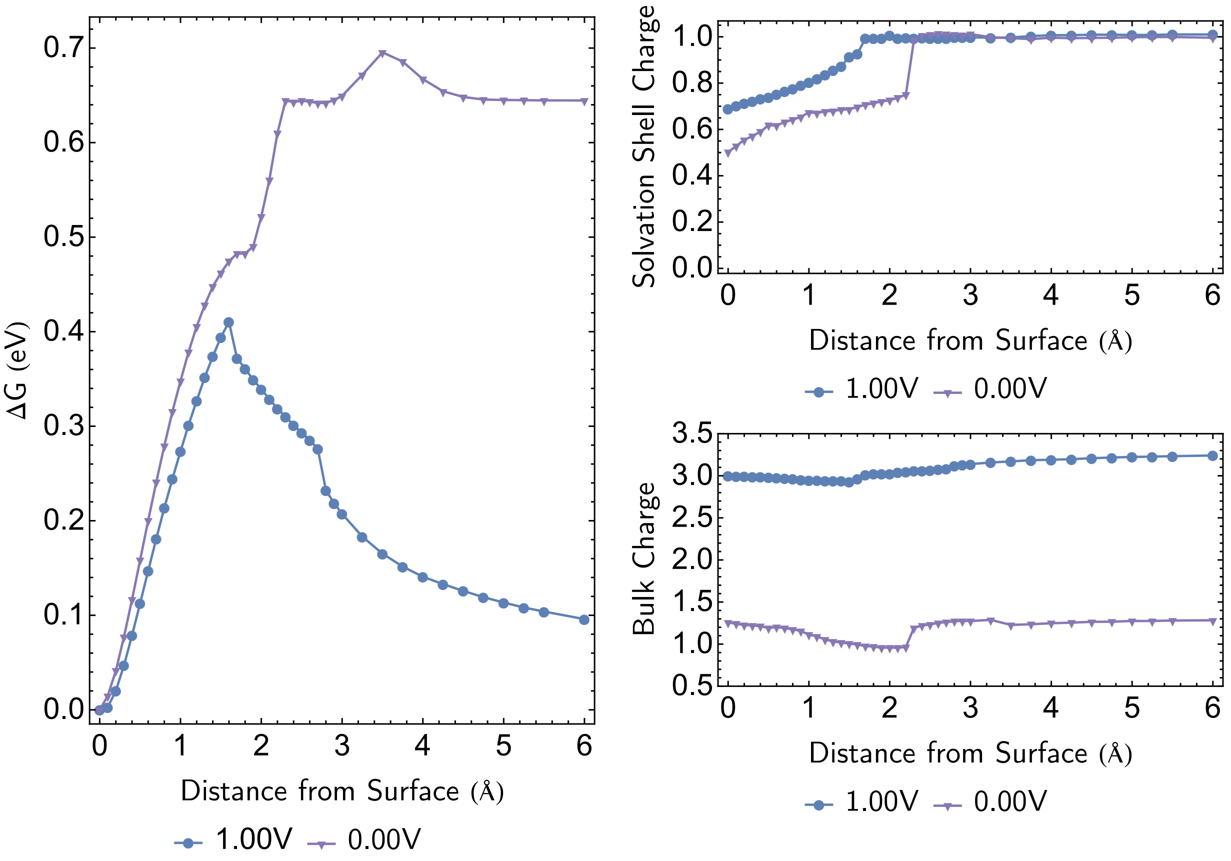

FIG. 11: Free energy curves and partial charge profiles for the half-etched corrosion (Ag_{28}) calculated at 0.00 V vs. SHE and 1.00 V vs, SHE. *(left)* Free Energy curves relative to the ion displacement δz. *(right)* Partial charges localized on the departing solvation shell and the bulk electrode. We note that sum of the bulk charges and the solvation shell charges for each distance point will lead to the charge on the total unit cell.

We extended the same CEP protocol to the half-etched surface (Ag_{28}) as the intermediate case between the perfectly-flat (Ag_{24}) and last-atom-bound (Ag_{25}) cases. Fig. 11 shows the resulting free energy curve and the charge profile, where the corrosion is initiated from the step site of the half-etched structure. Noticeably, no divergent pathways were observed for a single voltage. However, there were qualitatively different trajectories observed for the corroding pathway between 0.00 V and 1.00 V, manifested by the horizontal displacement of the departing ion, which will be shown in the subsequent subsection. Partial charge analysis also does not deviate from the previous observations obtained from Ag_{25} and Ag_{24}, with the model capturing both the generation of the Ag^+ as well as the the image charge response.

2. *Potential Dependence of the Drift in Ag_{28} Dissolution*

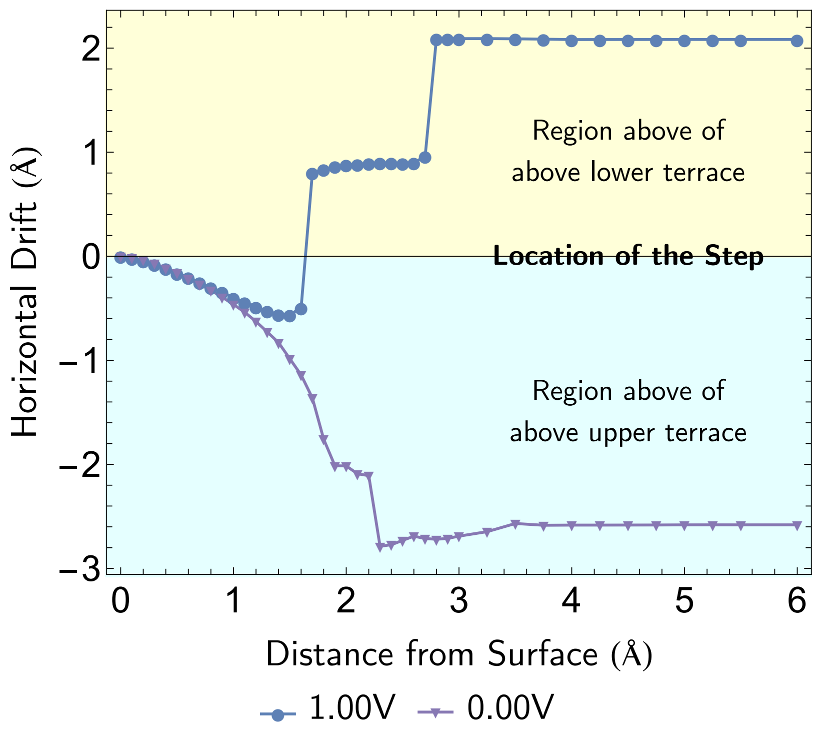

FIG. 12: Horizontal drift of the departing ion-solvation shell in the Ag_{28} corrosion. Identical to the case of Ag_{24}, the horizontal drift is confined to one-dimension, and the value can be plotted as a signed quantity to indicate the direction of the drift. For Ag_{28}, a 0 Å horizontal drift represents a vertical displacement above the location of the step. We note that values beyond 2.1 Å for the 0.00 V vs. SHE have been subtracted by the x-length of the model slab to better guide the reader, and this is valid considering the periodic boundary condition of the model.

For the case of Ag_{28} the departing species is more likely to drift towards the direction of the lower terrace of the step geometry when potential higher than the equilibrium is applied (Fig. 12) On the other hand, when potentials lower than the equilibrium is under effect, the departing ion is more likely for migrate towards the region above the upper terrace, forming a nanocluster-like structure. Schematic representation of this behavior if further visualized in Figure 13. Both of the observations align with the previously proposed mechanism that the first step towards Ag corrosion occurs through migration to a terrace site.[16]

3. *Thermodynamics of the Ag_{28} Case*

In Table V, the absolute free energies for uncorroded/corroded structures under strong potentials of 1.25 V and 1.50 V are provided for Ag_{28}. The results show that application of very strong potentials eventually leads a favorable corrosion, assuring the qualitative correctness of the CEP calculations on this model.

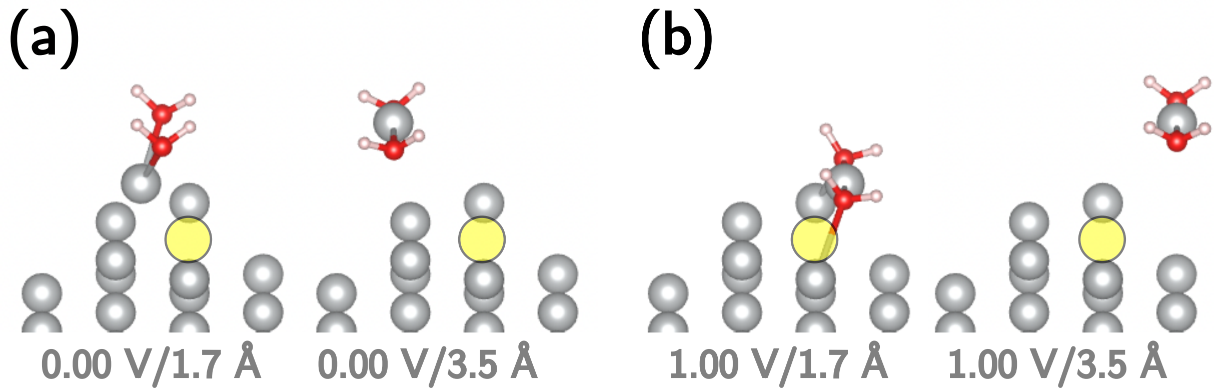

FIG. 13: Schematic of representing the horizontal drift with respect to the initial position of the departing Ag species (identified by yellow circle). (a) Drift of the departing ion-solvation shell under 0.00 V vs. SHE. The departing solvation shell is likely to "climb up" towards the higher terrace region due to stronger metal-metal interaction. Periodic boundary condition has been applied in the schematic representing 3.5 Å. (b) Drift of the departing ion-solvation shell under 1.00 V vs. SHE. The departing solvation shell is likely to drift towards the lower terrace region.

Potential (V)	G of Ag_{28} 0 Å sturcture (eV)	G of Ag_{28} 10 Å sturcture (eV)
1.25 V	-88.8536	**-88.9444**
1.50 V	-89.9813	**-90.2297**

TABLE V: **Absolute** free energies (G) for half-etched case of Ag_{28} for potentials higher than 1.00V. Energetically lower points are boldfaced.

V. NOTE 5: EMPIRICAL DFT DISPERSION CORRECTION

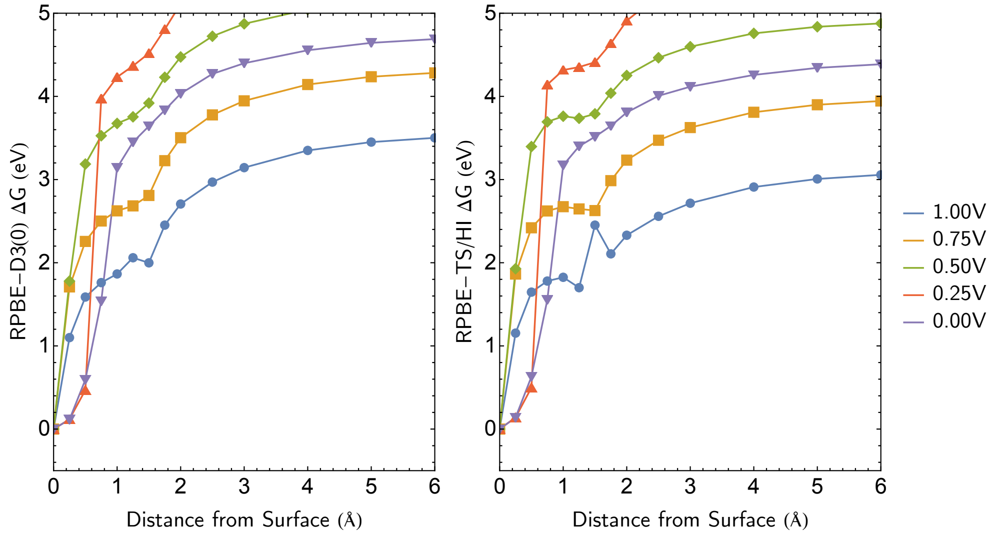

FIG. 14: Dispersion correction added free energy profiles. None of the methods presented captures the thermodynamics correctly and the free energy profiles are also erratic. (*left*) RPBE with Grimme's D3 dispersion correction with zero damping.[17] (*right*) RPBE with Tkatchenko-Scheffler dispersion correction with iterative Hirshfeld charge partitioning.[18,19]

Owing to its semilocal exchange-correlation functional, RPBE[20] cannot correctly capture long-range dispersion interactions such as van der Waals forces. Previous works have tested the use of adding non-local correlation[21] and utilizing a semilocal functional that directly involves the van der Waals corrections[8,22]. We tested using empirical DFT dispersion corrections onto our calculations, specifically the DFT-D3 with zero damping (*i.e.*, DFT-D3(0))[17] and Tkatchenko-Scheffler method with iterative Hirshfeld partitioning scheme (DFT-TS/HI)[18,19] by appending an additional single-point electronic energy calculation on the CEP optimized structures.

Interestingly, we observe that the addition of dispersion correction errs the energies and activation barriers by predicting corrosion to be thermodynamically unfavored even for the strongest potential bias in the case of Ag_{25}, overbinding the Ag atom to the surface (Figure 14). Noticeably, RPBE-TS/HI is less erratic given that the corroded geometry is approximately 0.5 eV lower in energy when compared to the case with RPBE-D3(0). We suspect that this is due to the adeptness of the DFT-TS/HI correction that accommodates for the fractionally charged atomic states that are necessary for CEP protocol, compared to the DFT-D3(0) correction which only accommodates for the neutral atomic reference states. Nevertheless, both free energy curves are still inaccurate which shows that the long-range correlation between surface and Ag atom (at a dissolution limit) is overestimated. Indeed, long-range dispersion interaction should be effectively mitigated due to solvent molecules, and artificially adding this contribution estimated from gas phase structures will overcount this interaction. This observation differs from previous studies that concluded the addition of non-local correlation to be important for correct chemisorption[21] or that concluded addition of van der Waals interaction does not affect the energy profile.[8]

VI. NOTE 6: SUPPLEMENTARY NOTE ON COMPARISON WITH PREVIOUS KINETIC STUDIES

It is useful to differentiate our experimental kinetic studies from previous ones in the literature. For example, Liu et al.[23] made measurements in ionic liquid, reporting rate constants of $10^{-4} - 10^{-5}$ cm/s and compared this to the value of $k_0 = 0.26 \pm 0.04$ cm/s from an earlier report by Gerischer.[24] While these studies are useful, we are not convinced that the analysis of Liu et al., or the original analysis by Gerischer, reports on the kinetics of the intrinsic interfacial ion transfer step. The analysis by Liu et al. relies on numerous assumptions buried in the analytical solutions developed for the transient deposition and stripping waves that cannot be verified experimentally. Both sets of measurements performed on macroscopic bulk polycrystalline silver electrodes whose surface nanostructure is likely roughening in uncontrolled ways during potential cycling and whose surfaces were not analyzed. The convoluted effects of surface roughening in Gerischer's study are supported by the observation of exchange current densities (4.5 ± 0.5 A/cm^2 and $\alpha = 0.74 \pm 0.02$ for 1 M $HClO_4$ + 0.1 M $AgNO_3$) that are two orders of magnitude greater than the values we obtained (42.0 $\pm$ 2.5 mA/cm^2 and $\alpha = 0.39 \pm 0.02$ for 4.7 M $AgNO_3$ at 22 °C). Gerischer et al. later published an additional study[16] that provides further insights into the nucleation/crystallization process by quantitatively discussing the adsorption of ad-atoms and the growth of crystals. Unfortunately, they did not present the kinetics parameter regarding the adjusted exchange current density or rate constants for us to make a comparison to. Given these substantial uncertainties regarding the surface structures and assumptions in the data analysis for these previous studies, compared with the precisely pre-prepared Ag metal clusters/nanoparticles on Au in our study, we do not think direct quantitative comparison of the kinetic parameters is useful.

VII. NOTE 7: SUPPORTING FIGURES FOR KINETIC MEASUREMENT OF ION TRANSFER IN SILVER ELECTRODISSOLUTION AND ELECTRODEPOSITION

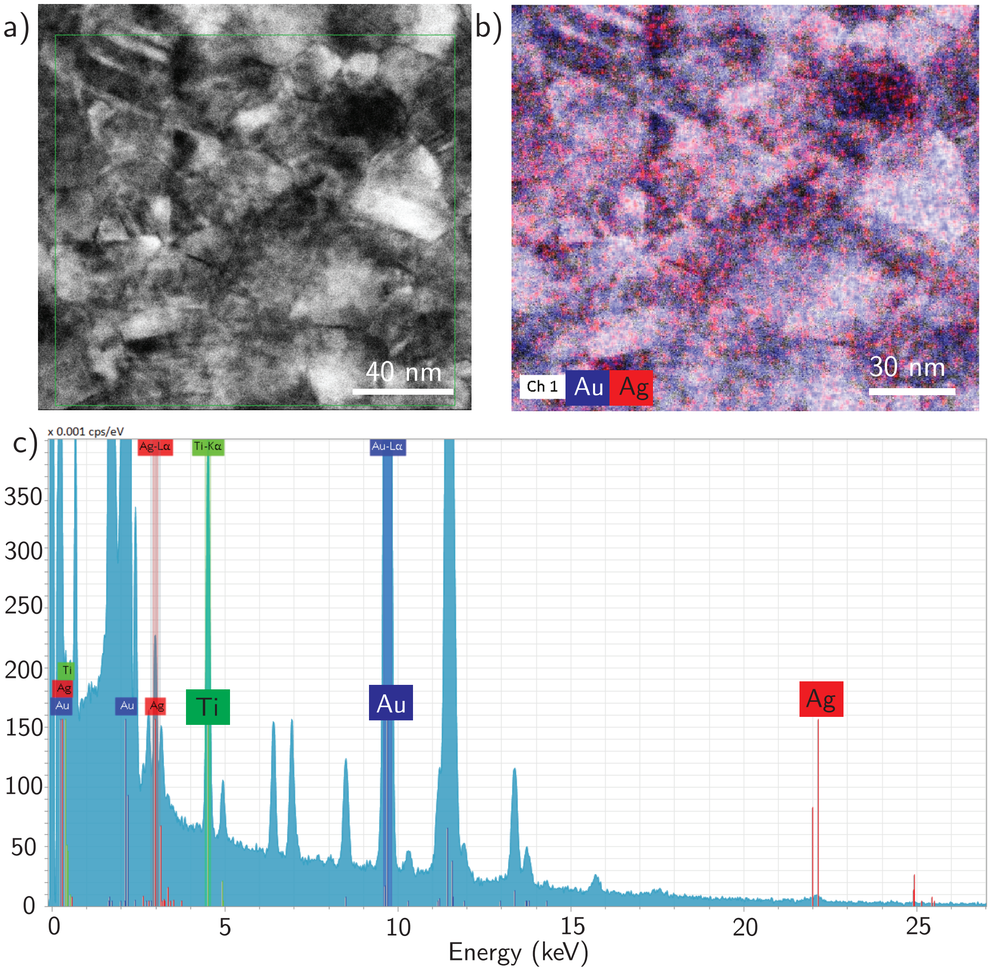

FIG. 15: High-resolution scanning transmission electron microscopy (HRSTEM) analysis of model Ag electrodes. (a) HRSTEM image of Ag nanoparticles supported by Au. (b) STEM-EDX element-mapping analyses with integrated values for Au (blue) and Ag (red) peaks demonstrating Ag nanoparticles as uniformly distributed and effectively isolated. (c) EDX elemental analysis of the Ti, Ag, and Au.

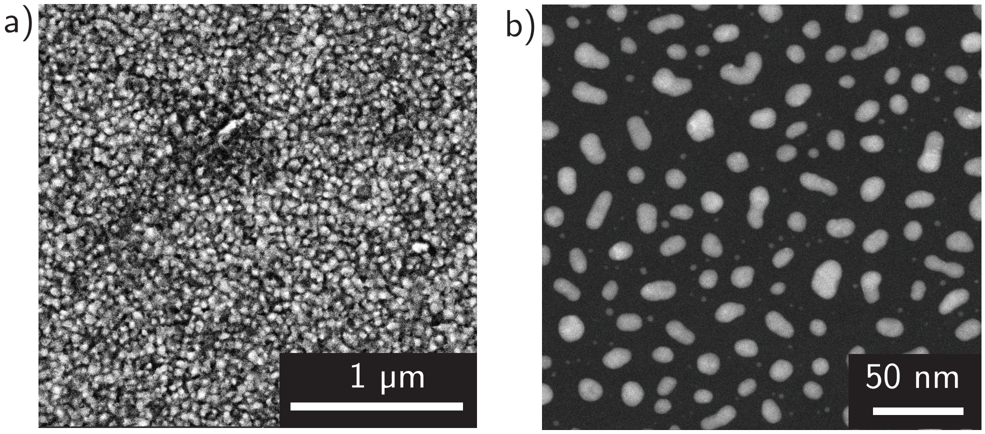

FIG. 16: (a) SEM image of the model Ag electrodes supported by Au. (b) SEM image of 2 nm Ag deposited without the support of 50 nm Au layer.

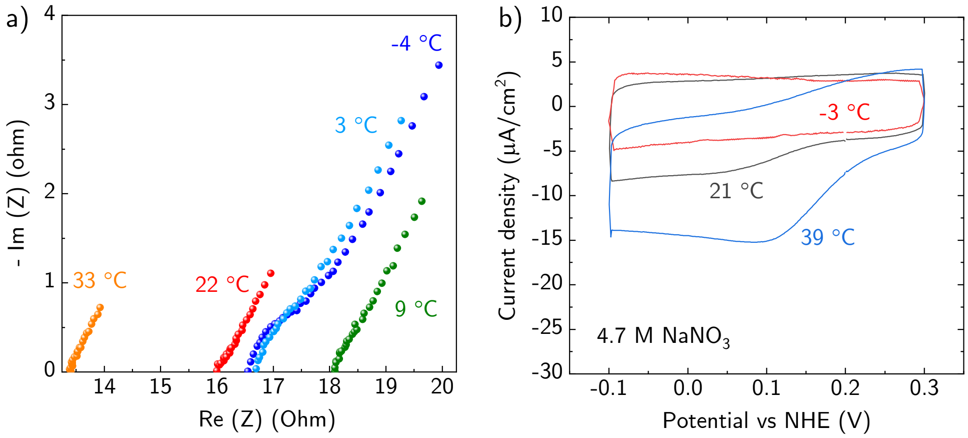

FIG. 17: (a) Nyquist plot of EIS responses at various temperatures in 4.7 M $AgNO_3$, using model Ag nanoparticles on Au working electrode. (b) Voltammograms collected at a rate of 50 mV/s in 4.7 M $NaNO_3$ using a model Ag nanoparticles on Au working electrode carried out at -3 °C, 21 °C and 39 °C, respectively to determine the initial duration required to complete the double-layer charging process. Regarding these Nyquist plots, we note that it is not possible to extract kinetic data from these impedance experiments because the kinetics are sufficiently fast that no semicircular RC element is evident. The high-frequency intercept with the x-axis is associated with the cell ohmic resistance, which generally decreases with increasing temperature, but also varies due to small differences in the electrode placement in the cell (each curve is a separate electrode). We conducted impedance measurements for each new Ag electrode and used the ohmic resistance only for correction for iR and RC time constants. All kinetic analysis was made by the potential step measurements described in the main text.

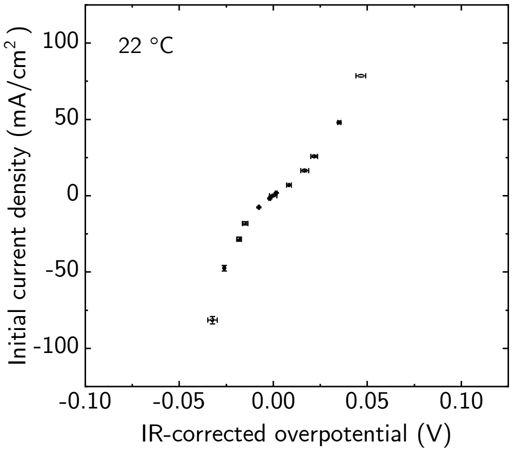

FIG. 18: (a) Repeated measurements were conducted – three at each overpotential – in a random sequence at temperature of 22 °C to validate reproducibility of experiments with minor variations denoted by aging of solution and replacement of Ag electrodes.

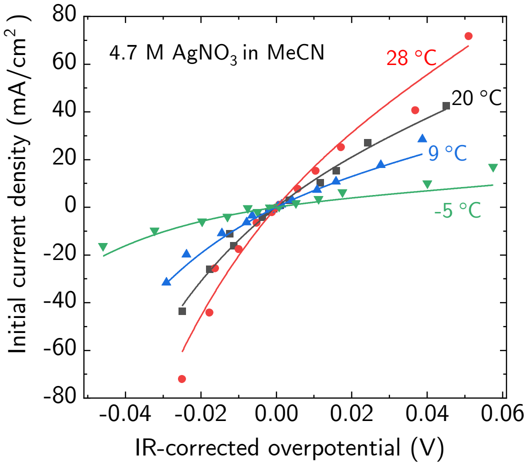

FIG. 19: Temperature- and driving force (overpotential)- dependent initial rates of Ag corrosion and deposition in acetonitrile system (0.1 % H_2O).

VIII. NOTE 8: SUPPLEMENTARY DISCUSSION ON THE MEASUREMENT ON MORE REACTIVE METALS

Here, we use Zn as an example to demonstrate the expansion of the methodology developed in this study to handle more reactive metals that are prone to spontaneous corrosion. Zn/Zn^{2+} has a redox potential of ≈ -0.76 V versus SHE. This indicates that within the electrochemical potential region used to investigate Zn deposition/dissolution, the hydrogen evolution reaction (HER) will take place spontaneously due to thermodynamic favorability. The experimental measurements should adhere to the same principles as we show for Ag, which involves using a small-cluster Zn electrode to ensure large numbers of active sites for dissolution and deposition avoiding nucleation barriers, a high concentration of Zn electrolyte to minimize concentration overpotentials, and deriving the initial current from transient curves.

In the cathodic Zn deposition process, the HER (hydrogen evolution reaction) will take place simultaneously as another cathodic reaction and competes with it. Modern techniques like rotating-ring disk electrode or online mass spectroscopy can be readily utilized to accurately and quantitatively assess the rate of HER in real-time, therefore enabling us to identify the rate of Zn deposition relative to HER. Furthermore, HER is significantly inhibited in the kinetics on the Zn electrode. In our experimental setup, which involves a high concentration of Zn ions and a Zn cluster electrode with numerous active sites, the instantaneous rate we obtained by extracting initial rates should be primarily attributed to the Zn deposition process. The impact of HER is expected to be minimal and can most likely be neglected in many cases.

In more-complicated scenarios with Zn dissolution process, Evans Diagrams that are experimentally determined via driving-force dependent measurements can be used. This diagram reveals the presence of microgalvanic cells. The resulting current is a combination of the cathodic process of the HER (hydrogen evolution reaction) and the anodic process of zinc dissolution. The current depends on the equilibrium potential, transfer coefficient, and exchange current density of the two independent half-reactions. We expect a significant decrease of the corrosion rate from enhancing the Zn kinetics by the utilization of concentrated Zn electrolyte and Zn cluster electrodes. The obtained Evans diagram can be further analyzed by separating the cathodic Tafel branch of the HER and the anodic Tafel branch of Zn. The Tafel branch of HER can be achieved by replacing Zn salts, such as $ZnCl_2$, with an anion of the same concentration, such as NaCl, in the same system configuration. Measurements to access the Tafel branch of HER can be performed in the potential region where Zn corrosion is inhibited. Consequently, the Tafel branch of Zn dissolution can be extracted and used for investigating the kinetics of ion transfer following the same approaches as in the main manuscript for Ag.

REFERENCES

[1] J. F. Janak, "Proof that $\partial e/\partial n_i = \varepsilon$ in density-functional theory," Phys. Rev. B **18**, 7165 (1978).

[2] J. P. Perdew, R. G. Parr, M. Levy, and J. L. Balduz Jr, "Density-functional theory for fractional particle number: derivative discontinuities of the energy," Phys. Rev. Lett. **49**, 1691 (1982).

[3] W. Yang, Y. Zhang, and P. W. Ayers, "Degenerate ground states and a fractional number of electrons in density and reduced density matrix functional theory," Phys. Rev. Lett. **84**, 5172 (2000).

[4] D. Hait and M. Head-Gordon, "Delocalization errors in density functional theory are essentially quadratic in fractional occupation number," J. Phys. Chem. Lett. **9**, 6280–6288 (2018).

[5] J. D. Goodpaster, A. T. Bell, and M. Head-Gordon, "Identification of possible pathways for C–C bond formation during electrochemical reduction of CO_2: New theoretical insights from an improved electrochemical model," J. Phys. Chem. Lett. **7**, 1471–1477 (2016).

[6] J. A. Gauthier, S. Ringe, C. F. Dickens, A. J. Garza, A. T. Bell, M. Head-Gordon, J. K. Nørskov, and K. Chan, "Challenges in modeling electrochemical reaction energetics with polarizable continuum models," ACS Catal. **9**, 920–931 (2018).

[7] J. A. Gauthier, C. F. Dickens, S. Ringe, and K. Chan, "Practical considerations for continuum models applied to surface electrochemistry," ChemPhysChem **20**, 3074–3080 (2019).

[8] J. A. Gauthier, Z. Lin, M. Head-Gordon, and A. T. Bell, "Pathways for the formation of C_{2+} products under alkaline conditions during the electrochemical reduction of CO_2," ACS Energy Lett. **7**, 1679–1686 (2022).

[9] R. Sundararaman, W. A. Goddard III, and T. A. Arias, "Grand canonical electronic density-functional theory: Algorithms and applications to electrochemistry," J. Chem. Phys. **146**, 114104 (2017).

[10] M. M. Melander, M. J. Kuisma, T. E. K. Christensen, and K. Honkala, "Grand-canonical approach to density functional theory of electrocatalytic systems: Thermodynamics of solid-liquid interfaces at constant ion and electrode potentials," J. Chem. Phys. **150**, 041706 (2019).

[11] S. Trasatti and E. Lust, "The Potential of Zero Charge," in *Modern Aspects of Electrochemistry*, edited by B. E. C. Ralph E. White, J. O'M. Bockris (Springer, 1999) Chap. 1, pp. 1–215.

[12] K. Momma and F. Izumi, "VESTA: a three-dimensional visualization system for electronic and structural analysis," J. Appl. Crystallogr. **41**, 2 (2008).

[13] K. Momma and F. Izumi, "Vesta 3 for three-dimensional visualization of crystal, volumetric and morphology data," J. Appl. Crystallogr. **44**, 1272–1276 (2011).

[14] D. R. Lide, *CRC handbook of chemistry and physics*, Vol. 85 (CRC press, 2004).

[15] G. Valette, "Double layer on silver single crystal electrodes in contact with electrolytes having anions which are slightly specifically adsorbed: Part ii. the (100) face," J. Electroanal. Chem. Interf. Electrochem. **138**, 37–54 (1982).

[16] H. Gerischer, "Zum mechanismus der elektrolytischen abscheidung und auflösung fester metalle ii. galvanostatische einschaltvorgänge an silberelektroden und die kinetik des kristallwachstums," Z. Elektrochem. Ber. Bunsenges. Phys. Chem. **62**, 256–264 (1958).

[17] S. Grimme, J. Antony, S. Ehrlich, and H. Krieg, "A consistent and accurate ab initio

parametrization of density functional dispersion correction (DFT-D) for the 94 elements H-Pu," J. Chem. Phys. **132**, 154104 (2010).
[18]T. Bucko, S. Lebegue, J. Hafner, and J. G. Angyan, "Improved density dependent correction for the description of London dispersion forces," J. Chem. Theory Comput. **9**, 4293–4299 (2013).
[19]T. Bučko, S. Lebègue, J. G. Ángyán, and J. Hafner, "Extending the applicability of the Tkatchenko-Scheffler dispersion correction via iterative Hirshfeld partitioning," J. Chem. Phys. **141**, 034114 (2014).
[20]B. Hammer, L. B. Hansen, and J. K. Nørskov, "Improved adsorption energetics within density-functional theory using revised perdew-burke-ernzerhof functionals," Phys. Rev. B **59**, 7413 (1999).
[21]A. J. Garza, A. T. Bell, and M. Head-Gordon, "Is subsurface oxygen necessary for the electrochemical reduction of CO_2 on copper?" J. Phys. Chem. Lett. **9**, 601–606 (2018).
[22]C. N. Lininger, J. A. Gauthier, W.-L. Li, E. Rossomme, V. V. Welborn, Z. Lin, T. Head-Gordon, M. Head-Gordon, and A. T. Bell, "Challenges for density functional theory: Calculation of CO adsorption on electrocatalytically relevant metals," Phys. Chem. Chem. Phys. **23**, 9394–9406 (2021).
[23]D. Liu, D. Krulic, H. Groult, and N. Fatouros, "Silver ion deposition on gold and silver disc electrodes from aqueous solutions and from dry or wet [emim][ntf2] room-temperature ionic liquid," J. Electroanal. Chem. **775**, 91–104 (2016).
[24]H. Gerischer and R. P. Tischer, "Zum mechanismus der elektrolytischen abscheidung und auflösung fester metalle i. vorgänge bei der stationären auflösung von silberelektroden und die geschwindigkeit der durchtrittsreaktion," Z. Elektrochem. **61**, 1159–1162 (1957), https://onlinelibrary.wiley.com/doi/pdf/10.1002/bbpc.19570610908.
